# Identification of ferroptosis-associated biomarkers for the potential diagnosis and treatment of postmenopausal osteoporosis

**DOI:** 10.3389/fendo.2022.986384

**Published:** 2022-08-29

**Authors:** Yunxiang Hu, Jun Han, Shengqiang Ding, Sanmao Liu, Hong Wang

**Affiliations:** ^1^ Department of Orthopedics, Dalian Municipal Central Hospital Affiliated of Dalian Medical University, Dalian, China; ^2^ School of Graduates, Dalian Medical University, Dalian, China; ^3^ Department of Spine Surgery, the First Affiliated Hospital of Dalian Medical University, Dalian, China; ^4^ Department of Spine Surgery, The People’s Hospital of Liuyang City, Changsha, China

**Keywords:** postmenopausal osteoporosis, ferroptosis, biomarker, consensus clustering, candidate genes

## Abstract

**Objective:**

Postmenopausal osteoporosis (PMOP) is one of the most commonly occurring conditions worldwide and is characterized by estrogen deficiency as well as persistent calcium loss with age. The aim of our study was to identify significant ferroptosis-associated biomarkers for PMOP.

**Methods and materials:**

We obtained our training dataset from the Gene Expression Omnibus (GEO) database using GSE56815 expression profiling data. Meanwhile, we extracted ferroptosis-associated genes for further analysis. Differentially expressed ferroptosis-associated genes (DEFAGs) between OP patients and normal controls were selected using the “limma” package. We established a ferroptosis-associated gene signature using training models, specifically, random forest (RF) and support vector machine (SVM) models. It was further validated in another dataset (GSE56814) which also showed a high AUC: 0.98, indicating high diagnostic value. Using consensus clustering, the OP patient subtypes were identified. A ferroptosis associated gene (FAG)-Scoring scheme was developed by PCA. The important candidate genes associated with OP were also compared between different ferrclusters and geneclusters.

**Results:**

There were significant DEFAGs acquired, of which five (HMOX1, HAMP, LPIN1, MAP3K5, FLT3) were selected for establishing a ferroptosis-associated gene signature. Analyzed from the ROC curve, our established RF model had a higher AUC value than the SVM model (RF model AUC:1.00). Considering these results, the established RF model was chosen to be the most appropriate training model. Later, based on the expression levels of the five DEFAGs, a clinical application nomogram was established. The OP patients were divided into two subtypes (ferrcluster A, B and genecluster A, B, respectively) according to the consensus clustering method based on DEFAGs and differentially expressed genes (DEGs). Ferrcluster B and genecluster B had higher ferroptosis score than ferrcluster A and genecluster A, respectively. The expression of COL1A1 gene was significantly higher in ferrcluster B and gencluster B compared with ferrcluster A and gencluster A, respectively, while there is no statistical difference in term of VDR gene, COL1A2 genes, and PTH gene expressions between ferrcluster A and B, together with gencluster A and B.

**Conclusions:**

On the basis of five explanatory variables (HMOX1, HAMP, LPIN1, MAP3K5 and FLT3), we developed a diagnostic ferroptosis-associated gene signature and identified two differently categorized OP subtypes that may potentially be applied for the early diagnosis and individualized treatment of PMOP. The ER gene, VDR gene, IL-6 gene, COL1A1 and COL1A2 genes, and PTH gene are important candidate gene of OP, however, more studies are still anticipated to further elucidate the relationship between these genes and ferroptosis in OP.

## Introduction

Osteoporosis (OP)occurs when bone metabolism fails, leading to low bone mineral density (BMD), microarchitecture deterioration, increased fragility in bones and subsequent susceptibility to fracture. OP is thought to result from an imbalance between bone resorption and bone synthesis of osteoclasts and osteoblasts (1, 2). Postmenopausal osteoporosis (PMOP) is one of the most commonly occurring conditions worldwide and is characterized by estrogen deficiency as well as persistent calcium loss with age (3). To prevent bone loss, a proactive approach is recommended to identify patients at high risk of PMOP. The development of high-throughput technologies has made gene microarray analysis a valuable method of identifying differentially expressed genes (DEGs) and thus, they could be used as biomarkers for various diseases. There have been several studies using gene microarrays to identify key genes involved in PMOP pathogenesis (4, 5). Combining multiple gene microarray analyses may help identify more accurate genetic biomarkers.

Ferroptosis is an iron-dependent form of regulated cell death driven by the lethal accumulation of lipid peroxidation. There is evidence that ferroptosis plays a role in the development and progression of many diseases, including cancer, necroinflammatory disorders, and many organ damages and degenerative changes that involve medicine, surgery, and even reproduction system (6–8). In addition, a growing body of evidence suggested that ferroptosis may represent as a new therapeutic target for osteoporosis (9–12). However, the potential biomarkers of ferroptosis-associated genes in the diagnosis and treatment of PMOP remain lacking. Therefore, in our study, DEFAGs were screened between OP patients and normal controls. Later, in order to predict the potential diagnostic value of DEFAGs in OP, we conducted a random forest (RF) analysis. On the basis of five explanatory variables (HMOX1, HAMP, LPIN1, MAP3K5 and FLT3), we developed a diagnostic ferroptosis-associated gene signature. We hypothesized that our signature would have a high diagnostic value, then we further validated it in another dataset (GSE56814). Finally, two subtypes of OP patients were categorized using consensus clustering, which might aid in the early diagnosis and individualized treatment of PMOP.

## Materials and methods

### Data retrieval

GSE56815 was downloaded from the (GEO) database (http://www.ncbi.nlm.nih.gov/geo) which contains data of 40 OP and 40 healthy control patients. GSE56814 containing 42 high BMD and 31 low BMD patients was downloaded for validation. All patients in both datasets were premenopausal or postmenopausal female and the sample tissues were derived from circulating monocytes. 382 ferroptosis-associated genes were retrieved from FerrDb (13) (**Supplementary Table S1**).

### Identification of DEFAGs

“limma” package was used to screen DEFAGs between 40 OP and 40 healthy control patients with an inclusion criteria p<0.05. heatmaps were plotted using the “pheatmap” package to describe the expression of DEFAGs between OP and healthy controls.

### Construction and validation of random forest model, and support vector machine model

RF and SVM models were built using the “randomForest” and “kernlab” R package, DEFAGs are used as explanatory variables, while OP patients are used as response variables. In order to obtain the most appropriate model for training, receiver operating characteristic (ROC), inverse cumulative distributions |residual|, and boxplots of |residual| were plotted. For selection of the explanatory variables, we ranked the explanatory variables by importance, and then the first five explanatory variables were selected. For further validation of our signature, GSE56814 containing 42 postmenopausal patients among which 16 were with OP and 26 were without OP was downloaded and analyzed.

### Construction of the nomogram model and machine learning for classification of PMOP and healthy woman

Based on the selected explanatory variables, a nomogram model was built using the “rms” package. We calculated every item’s score by projecting upward on a small scale (points) based on the characteristics of each variable of the patient. The total value was calculated by adding the scores of each item. A higher total value indicates a higher probability of OP. An analysis of the calibration curve, decision curve analysis (DCA) curve, as well as clinical impact curve were performed to test the accuracy of the model. Machine learning of genes to classify PMOP and healthy persons was performed. Following that, we reported the classification performance of the model using sensitivity, specificity, and an area under the receiver operator characteristic curve (14).

### Consensus clustering of DEFAGs

To investigate the role of DEFAGs in OP, the OP patients were divided into subgroups based on the expression of DEFAGs using “Consensus Cluster Plus” in R software. Groupings were based on the following principles (1): there is a small cumulative distribution function (CDF) that increases slowly; and (2) neither a small cluster nor a cross cluster exists in the cluster data.

### DEGs identification and functional annotation

DEGs between the different ferroptosis-associated subtypes were identified using the “limma” package in R with a fold-change of 1.5 and an adjusted p-value of < 0.05. GO and KEGG functional enrichment analyses were performed on the GO and KEGG datasets using the “clusterprofile” to identify gene functions and enriched pathways related to DEGs.

### Re-consensus clustering based on the DEGs

To further divide the OP patients into different subgroups based on the expression of DEGs, we used “Consensus Cluster Plus” in R software. Groupings were also based on the following principles (1): there is a small cumulative distribution function (CDF) that increases slowly; and (2) neither a small cluster nor a cross cluster exists in the cluster data.

### Construction of the FAG -scoring scheme and its clinical relevance

Principal component analysis (PCA) was used to develop a FAG-Scoring system that quantifies ferroptosis correlation levels in individual patients. The DEFAGs identified from OP and normal people were used. We then curated the expression profiles of the DEFAGs for PCA analysis, and we extracted the principal components 1 and 2 which acted as the signature score. Our FAG-Scoring scheme is calculated by adopting a formula similar to previous studies (15, 16): FAG-Score = ∑(PC1i+PC2i), where is the expression of phenotype-related genes determined from final ferroptosis.

### Statistical analysis

Statistical analysis was carried out using R software (version 3.6.2). For evaluating differences between the two independent groups, the student’s t-test was used (unpaired, two-tailed). Data from more than two groups were analyzed using one-way analysis of variance (ANOVA) and Kruskal-Wallis test as parametric and non-parametric methods, respectively. A P value < 0.05 was considered significant if not otherwise specified.

## Results

### Landscape of 45 DEFAGs in OP

**Figure 1:**
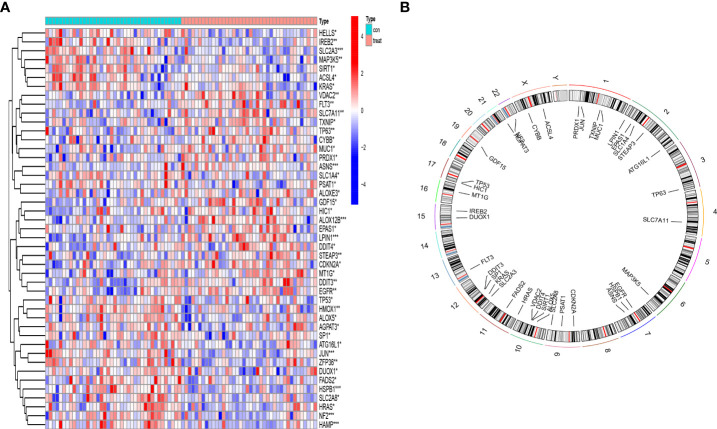
Landscape of 45 differentially expressed ferroptosis-associated genes (DEFAGs) in OP patients. **(A)** Heat map showing the expression of 45 DEFAGs between OP patients and healthy controls. **(B)** Chromosomal positions of the 45 DEFAGs were shown in the loop graph. *P < 0.05; **P < 0.01; ***P < 0.001.

Based on the inclusion criteria log2FC > 0.2 and p * 0.001, 211 differentially expressed genes (DEGs) between OP patients and normal controls were identified. Crossing the 211 DEGs with 382 ferroptosis-associated genes, 45DEFAGs were acquired (**Supplementary Table S2**). The 45 DEFAGs were expressed differently in OP patients and in healthy controls (**Figure 1A**). The chromosomal positions of the 45 DEFAGs were plotted on a loop graph (**Figure 1B**).

### Construction and validation of the RF model

**Figure 2:**
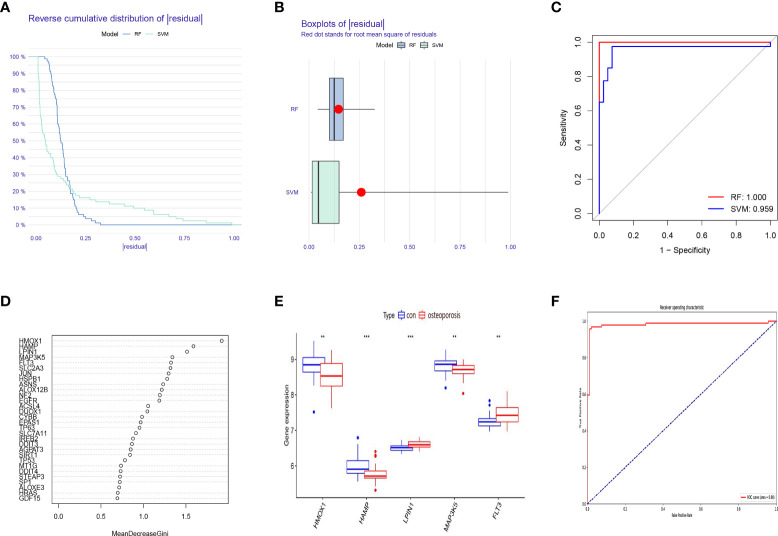
Construction and validation of a diagnostic ferroptosis-associated gene signature. **(A)** Cumulative distribution of |residual| in RF model and SVM model. **(B)** Boxplots of |residual| in RF model and SVM model. **(C)** ROC curve and AUC value of RF model and SVM model. **(D)** The relative importance of explanatory variables ranked by the RF model. **(E)** The comparison of these five genes between OP and normal patients. **(F)** The ROC result of our proposed signature in validation dataset. **P < 0.01; ***P < 0.001.

A RF model and SVM model were established independently to determine a diagnostic ferroptosis gene signature. Based on an analysis of the reverse cumulative distribution of |residual| and boxplots of |residual|, it is found that the RF model sustains the lowest residual distribution when compared to the SVM model (**Figures 2A, B**). Analyzed from the overall ROC curve, our established RF model had a higher AUC value than the SVM model (**Figure 2C**). Considering these results, the established RF model was chosen to be the most appropriate training model. We then further ranked the explanatory variables by importance (**Figure 2D**). We identified the first five explanatory variables (HMOX1, HAMP, LPIN1, MAP3K5, FLT3) and used them to establish a diagnostic ferroptosis associated gene signature (**Table 1**). ROC results for these 5 genes using the machine learning algorithms were showed in (**Supplementary Figure S1**). It was showed that there was statistical difference comparing these five genes between OP and normal patients (**Figure 2E**). For further validation of our signature, GSE56814 containing 42 postmenopausal patients among which 16 were with OP and 26 were without OP was analyzed. The ROC showed that this signature had a high AUC:0.98, indicating high diagnostic value (**Figure 2F**).

**Table:** Table 1 Basic information of these five genes.

gene	logFC	p-Value	adjusted p-Value	gene description	location
HMOX1	-0.29081	0.001245	0.020145	Heme oxygenase	chr22:35,380,361-35,394,214
HAMP	-0.22921	8.90E-05	0.007919	Hepcidin, iron homeostasis	chr19:35,280,716-35,285,143
MAP3K5	-0.13902	0.003441	0.04654	Mitogen-activated protein kinase kinase kinase 5	chr6:136,557,046-136,793,091
FLT3	0.195708	0.001245	0.020145	Receptor-type tyrosine-protein kinase	chr13:28,003,274-28,100,592
LPIN1	0.095823	7.14E-05	0.007919	Phosphatidate phosphatase	chr2:11,677,544-11,827,409

### Construction of the nomogram model and machine learning for classification of PMOP and healthy woman

**Figure 3:**
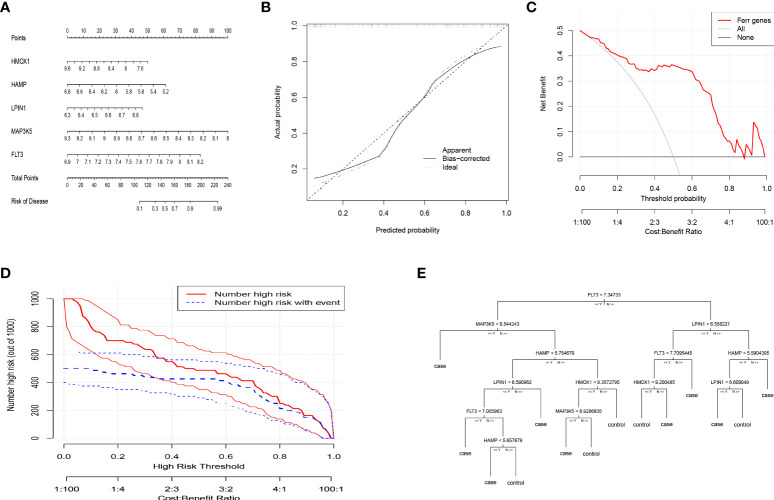
Construction of the nomogram model and Machine Learning for Classification of PMOP and Healthy Woman. **(A)** Construction of the nomogram model based on the selected DEFAGs (HMOX1, HAMP, LPIN1, MAP3K5, FLT3). **(B)** Calibration curve illustrating the diagnostic ability of the nomogram model. **(C)** Nomogram models have higher clinical utility than individual DEFAGs, according to DCA. **(D)** The clinical impact curve demonstrates a high level of diagnostic ability of the nomogram model. **(E)** Decision-tree diagram showed the results of gene expression levels that determine PMOP or a healthy woman.

To facilitate the diagnosis of PMOP for physicians using the selected DEFAGs (HMOX1, HAMP, LPIN1, MAP3K5, FLT3), a nomogram model was constructed (**Figure 3A**). Using the calibration curves, it was found that the nomogram model accurately predicted the positive rate of OP (**Figure 3B**). DCA showed that although both the nomogram model and individual DEFADs generated net benefits, the nomogram model generated significantly greater net benefits than the individual DEFADs, indicating that the nomogram model may be more clinically useful than individual DEFADs (**Figure 3C**). An analysis of the clinical impact curve suggests that the nomogram model has a relatively high diagnostic ability (**Figure 3D**). Step-by-step process showing how to identify a woman with PMOP versus a healthy woman was shown in (**Figure 3E**).

### Identification of OP subgroups by consensus clustering

**Figure 4:**
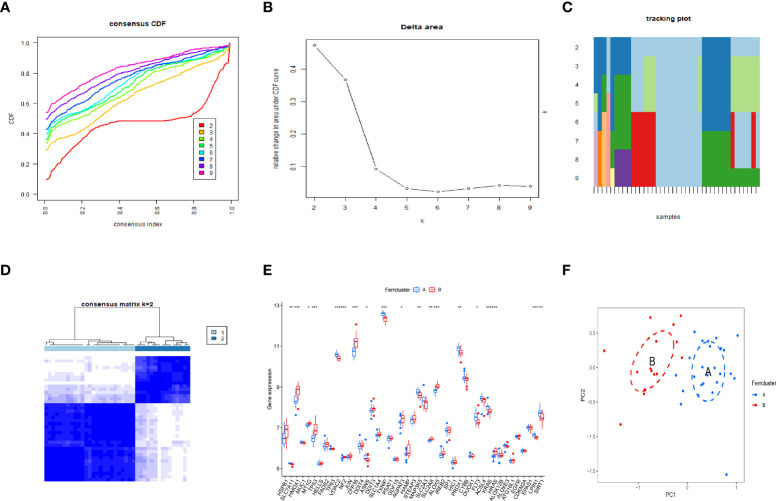
Two OP subgroups obtained by consensus clustering. **(A)** Curve of the CDF when k=2-9. **(B)** The delta area score of the CDF curve when k= 2–9. **(C)** Tracking plot when k =2–9. **(D)** The matrix heat map was neatly categorized when k=2. **(E)** The expression of 45 DEFAGs between ferrcluster A and ferrcluster B is shown in the histogram. **(F)** PCA based on the expression of the 45 DEFAGs can distinguish OP patients in ferrcluster A and ferrcluster B. *P < 0.05; **P < 0.01; ***P < 0.001.

By using the consensus clustering method, we divided the OP patients into several subgroups to investigate the role of the 45 DEFADs in OP. Based on the results, when k=2, 3, and 4, the CDF value increases gradually, and when k=4, the CDF value is small. (**Figures 4A, B**). There was a high correlation between groups, however, when OP patients were divided into three or four groups. In this regard, two subgroups (ferrcluster A and B) of patients with OP were analyzed (**Figures 4C, D**). **Figures 4E** shows that HSPB1, HMOX1, TP53, etc. were upregulated in the ferrcluster B compared with the ferrcluster A, whereas HRAS, LPIN1, SIRT1, etc. were downregulated (**Figures 4E**). To check the accuracy of our classification, PCA analysis based on the expression profiles of the 45 DEFAGs was performed, the result showed that it can effectively distinguish the ferrcluster A from the ferrcluster B (**Figures 4F**).

### DEGs identification and functional annotation

**Figure 5:**
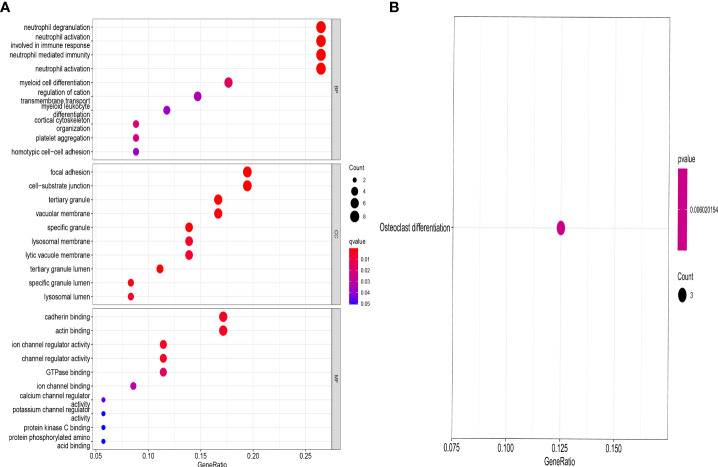
GO and KEGG enrichment analysis of DEGs. GO **(A)** and KEGG **(B)** showing functional enrichment of DEGs between the genecluster A and genecluster B.

DEGs between the different ferroptosis-associated subtypes were identified using the “limma” package in R with a fold-change of 1.5 and an adjusted p-value of <0.05. 37 DEGs were identified (**Supplementary Table S3**). GO and KEGG analysis were performed to investigate the DEGs. GO revealed that the patients in the DEGs were mainly enriched in processes such as neutrophil degranulation, neutrophil activation involved in immune response, focal adhesion, cell−substrate junction, tertiary granule, cadherin binding, actin binding, ion channel regulator activity. (**Figure 5A**). KEGG revealed that DEGs were mainly enriched in osteoclast differentiation (**Figure 5B**).

### Further identification of OP subgroups by consensus clustering based on DEGs

**Figure 6:**
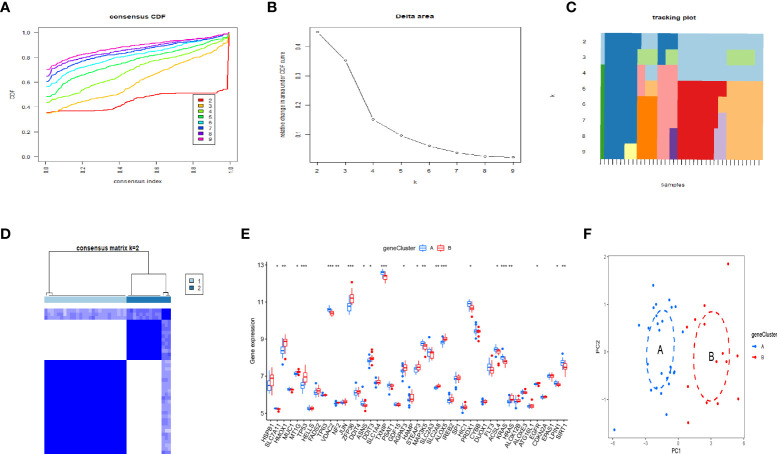
Two OP subgroups re-obtained by consensus clustering based on DEGs. **(A)** Curve of the CDF when k=2-9. **(B)** The delta area score of the CDF curve when k= 2–9. **(C)**Tracking plot when k =2–9. **(D)** The matrix heat map was neatly categorized when k=2. **(E)** The expression of 37 DEGs between the genecluster A and genecluster **(F)** PCA based on the expression of the 37 DEGs can distinguish OP patients in genecluster A and genecluster B. *P < 0.05; **P < 0.01; ***P < 0.001.

By using the consensus clustering method, we further divided the OP patients into several subgroups to investigate the role of the 37 DEGs in OP. Based on the results, when k=2, 3, and 4, the CDF value increases gradually, and when k=4, the CDF value is small (**Figures 6A, B**). There was a high correlation between groups, however, when OP patients were divided into three or four groups. In this regard, two subgroups of patients with OP were analyzed (**Figures 6C, D**). **Figure 6E** showed that HMOX1, TP53, ZFP36, etc. were upregulated in the genecluster B group compared with the genecluster A group, whereas KRAS, LPIN1, SIRT1, etc. were downregulated (**Figures 6E**). PCA analysis based on the expression profiles of the 37 DEGs was performed, the result showed that it can effectively distinguish the genecluter A from the genecluter B (**Figure 6F**).

### Construction of the FAG-scoring scheme and its clinical relevance

**Figure 7:**
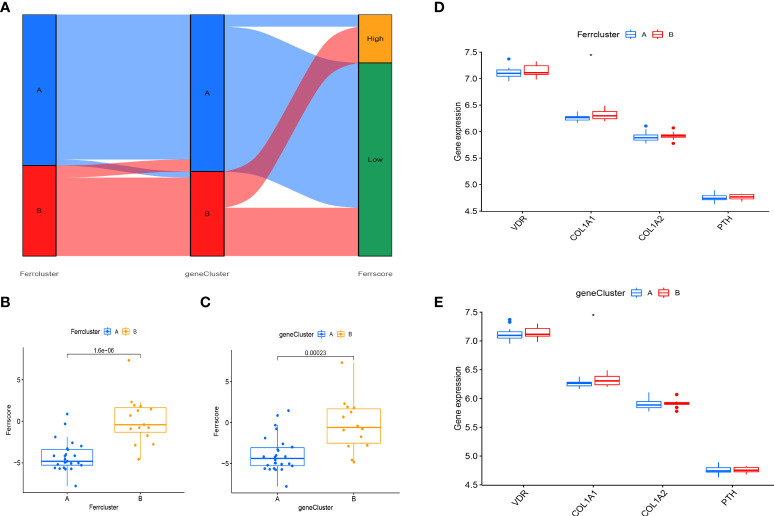
Construction of a FAG-Scoring system. **(A)** Alluvial diagram of subtype distributions in ferroptosis scores with different ferrclusters and geneclusters. **(B)** Comparison of ferroptosis scores between the ferrcluster A and ferrcluster A and B. **(C)** Comparison of ferroptosis score between the genecluster A and genecluster A and genecluster B. **(D)** The expression of VDR gene, COL1A1gene, COL1A2gene and PTH gene between the ferrcluster A and ferrcluster A and ferrcluster B. **(E)** The expression of VDR gene, COL1A1gene, COL1A2gene and PTH gene between the genecluster A and genecluster B. *P < 0.05.

Principal component analysis (PCA) was used to develop a FAG-Scoring system that quantifies ferroptosis correlation levels in individual patients. The DEFAGs identified from OP and normal people were used. We found that ferroptosis is involved in both ferrcluster A, B and genecluster A, B. Meanwhile, it is showed that ferrcluster B and genecluster B had higher ferroptosis score indicating a higher relevance to ferroptosis. Alluvial diagram of subtype distributions in ferroptosis scores with different ferroclusters and geneclusters was also performed (**Figure 7A**). Ferroptosis score of these DEFADs between ferrcluster A and ferrcluster B were compared. It is showed that ferrcluster B had a higher ferroptosis score than ferrcluster A (**Figure 7B**). Ferroptosis score of these DEFADs between genecluster A and genecluster B were also compared. It is showed that genecluter B had a higher ferroptosis score than genecluster A (**Figure 7C**). We also compared the important candidate genes associated with OP between ferrcluster A, B and gencluster A, B, respectively. It is showed that the expression of COL1A1 gene was significantly higher in ferrcluster B and gencluster B compared with ferrcluster A and gencluster A, respectively, while there is no statistical difference in term of VDR gene, COL1A2 genes, and PTH gene expressions between ferrcluster A and B, together with gencluster A and B (**Figures 7D, E**).

## Discussion

The OP can be categorized into two major categories: primary and secondary. Primary osteoporosis is divided into PMOP (type I), which generally occurs within 5-10 years after menopause in women; Senile OP (type II), which generally occurs after the age of 70; and idiopathic OP, which mainly occurs in adolescents and has an unknown etiology. Secondary OP is defined as OP caused by any disease and/or medication that affects bone metabolism and other definite causes (17). Postmenopausal women are at high risk for OP, and early screening and identification of those risk factors should be enhanced and preventive measures should be actively taken, even if a fragility fracture has already occurred (18). Thus early, molecular-level diagnosis and intervention in postmenopausal patients with OP is of profound clinical importance. OP can be prevented and treated more effectively with ferroptosis regulation, which is expected to have fewer side effects (19–21). It is still necessary to investigate the mechanism, as well as the signaling pathways related to ferroptosis to identify new treatment targets for OP. In our study, we detected that 45 DEFAGs (such as HSPB1, SLC7A11, HMOX1, TP53, JUN, HAMP, MAP3K5, SLC2A3, EGFR, LPIN1, SIRT1) between OP patients and healthy controls. In the next step, RF, and SVM models were developed separately in order to construct a ferroptosis-associated gene signature using 45 DEFAGs. RF refers to a learning algorithm that incorporates different decision trees. A RF model consists of independent sets of decision trees. Each tree is generated based on random samples. Every decision tree learns and makes predictions independently, and the final result is based on the mean value of all trees (22, 23). SVMs are discriminant classifiers that use a classification hyperplane to define their classification. Using labeled training samples, the model is trained, and then test samples are classified using the optimal hyperplane’s output (24, 25). Comparing the RF model with the SVM models, the established RF model sustains the lowest residual distribution and the highest AUC value, making it the most appropriate training model. Several diagnostic ferroptosis genes were identified, and a nomogram model, which incorporates five explanatory variables (HMOX1, HAMP, LPIN1, MAP3K5 and FLT3), was eventually developed. The analysis of DCA curve and a clinical impact curve suggested that the nomogram model is greatly beneficial for clinical diagnosis. Deng et al. (26) used GSE2208 and GSE56815 datasets for analysis and validation, they identified FOS, PTPN6, and CTSD as potential biomarkers for postmenopausal osteoporosis, which were different from ours. Using multiple osteoporosis microarray datasets (GSE2208, GSE56814, and GSE56815), Zhang et al. (27) identified and systematically characterized BMD-related genes, however, no validation was performed. Based on pathway analysis, Zhou et al. (28) identified five key independent pathways involved in regulating BMD that can be corrected for crosstalk effects providing an insight into osteoporosis functional networks. Under accession number GSE56814, the raw microarray data from this cohort has been deposited with GEO. Therefore, to further validate our signature, dataset (GSE56814) was used, notably, the results showed that in PMOP patients, our proposed signature had a high AUC: 0.98, indicating high diagnostic value. Using the nomogram generated by five explanatory variables, we could potentially select and treat PMOP patients at a comparatively early stage, and as it has been reported that early preventive treatment such as physical sports interventions, calcium and vitamin D supplements would significantly reduce the possibility of fragile fracture (3, 29). As part of exploring the role of 45 DEFAGs in OP, “ConsensusClusterPlus” was used to divide OP patients into two subgroups based on the expression of 45 DEFAGs. HSPB1, HMOX1, TP53, etc. were upregulated in the ferrcluster B group compared with the ferrcluster A group, whereas HRAS, LPIN1, SIRT1, etc. were downregulated. With a PCA analysis based on expression profiles of 45 DEFAGs, the ferrcluster A group can be distinguished from the ferrcluster B group. Our results indicate that there are two completely different subtypes of OP patients. We further identified DEGs between these two subgroups. GO revealed that the DEGs were mainly enriched in processes such as in neutrophil degranulation, neutrophil activation involved in immune response, focal adhesion, cell−substrate junction, tertiary granule, cadherin binding, actin binding, ion channel regulator activity. KEGG revealed that the DEGs were mainly enriched in osteoclast differentiation. Further studies could be performed to investigate the roles of DEGs in these enriched processes causing OP. We later constructed a FAG-Scoring system and our results found that ferroptosis is involved in both ferrcluster A, B and genecluster A, B. Meanwhile, it is showed that ferrcluster B and genecluster B had higher ferroptosis score indicating a higher relevance to ferroptosis.

PMOP is one category of the OP. OP is most likely a polygenetic disease, and these genes are closely linked to bone formation during bone growth and bone remodeling during bone loss (30). ER estrogen receptor (ER) genes, vitamin D receptor (VDR) genes, interleukin-6 (IL-6) genes, collagen type I (COL1A1 and COL1A2) genes, and parathyroid hormone (PTH) genes are among the most studied candidates in recent years (31–34). ER estrogen exerts its biological effects mainly through binding to the intranuclear specific receptors of osteoblasts and osteoclasts. There are three main types of ER have been identified: estrogen receptor α (ERα), estrogen receptor β (ERβ), and the G protein-coupled estrogen membrane receptor GPR30/GPER1 (35). Estrogen binds to receptors in osteoblasts, prompting them to secrete collagenase and release cytokines and growth factors for bone reconstruction. The effect of ER on osteogenesis is mediated by certain cytokines, such as insulin-like growth factor I (IGF-1) and tumor growth factor (TGF-β). Saoji et al. (36) studied the relationship between ER gene Pvu II and XbaI polymorphisms and bone mineral density in Indian women before and after menopause, and logistic analysis based on age, body mass index, and tobacco and alcohol consumption showed that the xx genotype in the XbaI gene polymorphism was associated with reduced spinal bone mass in postmenopausal women, suggesting that the X allele is protective. VDR gene polymorphisms can interfere with its mRNA expression and splicing, affecting the quantity and stability of mRNA, thus causing small differences in the level and function of VDR protein, which further affects bone metabolism through trans-activation between VDR protein and its target genes. Ahmad et al. (37) investigated the effect of vitamin D levels and bone mineral density in 508 postmenopausal women with OP in northern India by using genetic polymorphisms (BsmI and FokI) variant patterns of VDR, and showed that BMD was significantly lower in the spine and hip in bb genotype menopausal women, and in the spine, hip, femoral neck and forearm in ff genotype menopausal women. It is suggested that Bsm I and Fok I polymorphisms of VDR gene are significantly associated with low BMD and may be an important risk factor for OP. The study showed that estrogen works through IL-6 as a mediator against OP (38); Estrogen acts on the proximal 225bq sequence of the IL-6 gene promoter through the ER to inhibit IL-6 gene expression; in addition, estrogen modulates the signaling of gp130 (signal transducer), thereby altering the sensitivity of cells to IL-6 (39). COL1A1 and COL1A2 proteins are important proteins of bone and are the main components of bone organic matter, accounting for about 80% to 90% of bone matrix proteins, which play an important role in maintaining the integrity of bone structure, biomechanical properties and bone toughness. Grant et al. (40) was the first to report polymorphisms arising from a G→T mutation at the site on intron 1 of the COL1A1 gene that binds to the transcription factor Sp1: SS, Ss, ss, with a significantly higher incidence in OP patients (48%) than in normal subjects (20%); The frequency of COL1A1 polymorphism, especially the distribution of Sp1 binding site polymorphism, varied greatly among different races. Despite the racial variability of this gene polymorphism, the COL1A1 polymorphism has a greater value as a predictor of OP and fracture risk from a clinical perspective. However, there are no studies showing that polymorphic loci of COL1A2 gene are associated with OP. PTH as a bone resorption inhibitor is beneficial in the treatment of OP, however, PTH is a “double-edged sword” in the metabolism of bone (41). Small doses of PTH stimulate osteoblasts secreting collagen to form new bone, but large doses inhibit osteoblasts. A related study showed that continuous PTH injection stimulates osteoclast activity and reduces bone mass, whereas intermittent PTH injection stimulates osteoblast differentiation and increases bone mass (42). The ER gene, VDR gene, IL-6 gene, COL1A1 and COL1A2 genes, and PTH gene are important candidate genes associated with OP. The genetic basis of OP can be revealed through the study of OP candidate gene polymorphisms, which can identify genes closely related to OP. In our study, we further compared the important candidate genes associated with OP between ferrcluster A, B and gencluster A, B, respectively. It is showed that the expression of COL1A1 gene was significantly higher in ferrcluster B and gencluster B compared with ferrcluster A and gencluster A, respectively, while there is no statistical difference in term of VDR gene, COL1A2 genes, and PTH gene expressions between ferrcluster A and B, together with gencluster A and B. OP is a polygenic and complex disease, and future studies should pay attention to the gene-to-gene association, and multiple genes should be considered comprehensively in exploring the mechanism of OP, so as to provide a genetic basis for early clinical screening of OP patients and determination of high-risk groups for fracture, this will facilitate early and effective prevention and treatment of OP. Current research on OP has focused on iron overload and reactive oxygen species (ROS) production, but the intrinsic link between ferroptosis and OP has not been thoroughly investigated (43). Chinese herbal medicines are potentially valuable in the treatment of OP, and herbal components (artesunate (44), quercetin (45), etc. can regulate ferroptosis, but their specific mechanisms of action are not yet clear. The role of other herbal monomers in the regulation of ferroptosis needs to be further explored. In conclusion, an in-depth study of ferroptosis can help further analyze the pathogenesis of OP and provide a basis for the prevention and clinical treatment of OP.

In future studies, through differential analysis, we wish to gather more clinical characteristics such as gender, BMI, history of smoking, BMD findings that will help us distinguish between these two completely different subtypes of PMOP, which might pave the way for future diagnosis and targeted treatment of PMOP.

## Limitations

The study had certain limitations. Firstly, the sample tissues were derived from circulating monocytes rather than bone tissues (osteoclasts and osteoblasts), As a result, we could not determine if the selected diagnostic markers would be appropriate to measure in bone tissue (osteoclasts and osteoblasts). Secondly, basic science and clinical studies are needed to validate our findings. At this point, our research results are only predictions.

## Conclusion

On the basis of five explanatory variables (HMOX1, HAMP, LPIN1, MAP3K5 and FLT3), we developed a diagnostic ferroptosis-associated gene signature and identified two differently categorized OP subtypes that may potentially be applied for the early diagnosis and individualized treatment of PMOP. The ER gene, VDR gene, IL-6 gene, COL1A1 and COL1A2 genes, and PTH gene are important candidate gene of OP, however, more studies are still anticipated to further elucidate the relationship between these genes and ferroptosis in OP.

## Data availability statement

The original contributions presented in the study are included in the article/**Supplementary Material**. Further inquiries can be directed to the corresponding author.

## Author contributions

HW contributed to study conception and design. YH, JH and SD collected, analyzed clinical data and wrote the manuscript. YH and SL were involved in submitting and revising the paper. The final version of manuscript was read and approved by all authors.

## Funding

This work was funded by the National Natural Science Foundation of China (grant number: 31971275).

## Conflict of interest

The authors declare that the research was conducted in the absence of any commercial or financial relationships that could be construed as a potential conflict of interest.

## Publisher’s note

All claims expressed in this article are solely those of the authors and do not necessarily represent those of their affiliated organizations, or those of the publisher, the editors and the reviewers. Any product that may be evaluated in this article, or claim that may be made by its manufacturer, is not guaranteed or endorsed by the publisher.

## Supplementary material

Supplementary Figure 1 ROC results for these 5 genes. (A-E) ROC results of HMOX1, HAMP, LPIN1, MAP3K5 and FLT3.

Supplementary Table 1 Identified 382 ferroptosis-associated genes.

Supplementary Table 2 Acquired 45DEFAGs.

Supplementary Table 3 Identified 37 DEGs.
