# Supplementary figures and images for: Identification of ferroptosis-associated biomarkers for the potential diagnosis and treatment of postmenopausal osteoporosis
Source: Front Endocrinol (Lausanne). 2022 Aug 29;13:986384. doi: 10.3389/fendo.2022.986384 (PMC9464919; doi:10.3389/fendo.2022.986384)

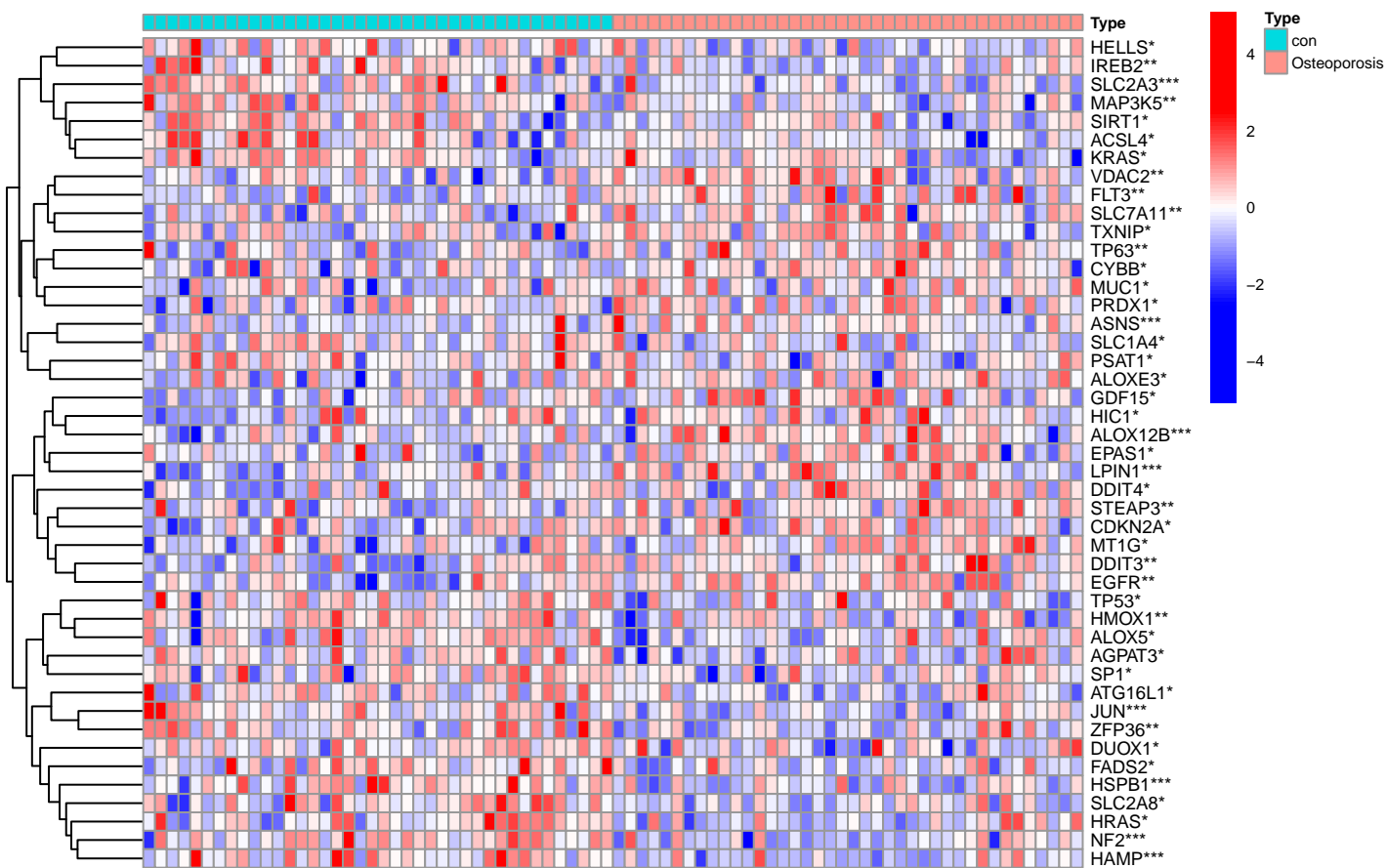

Supplement: Supplementary file 1 [file DataSheet_4.zip › Original data an R codes 1/08.diff/heatmap.pdf]

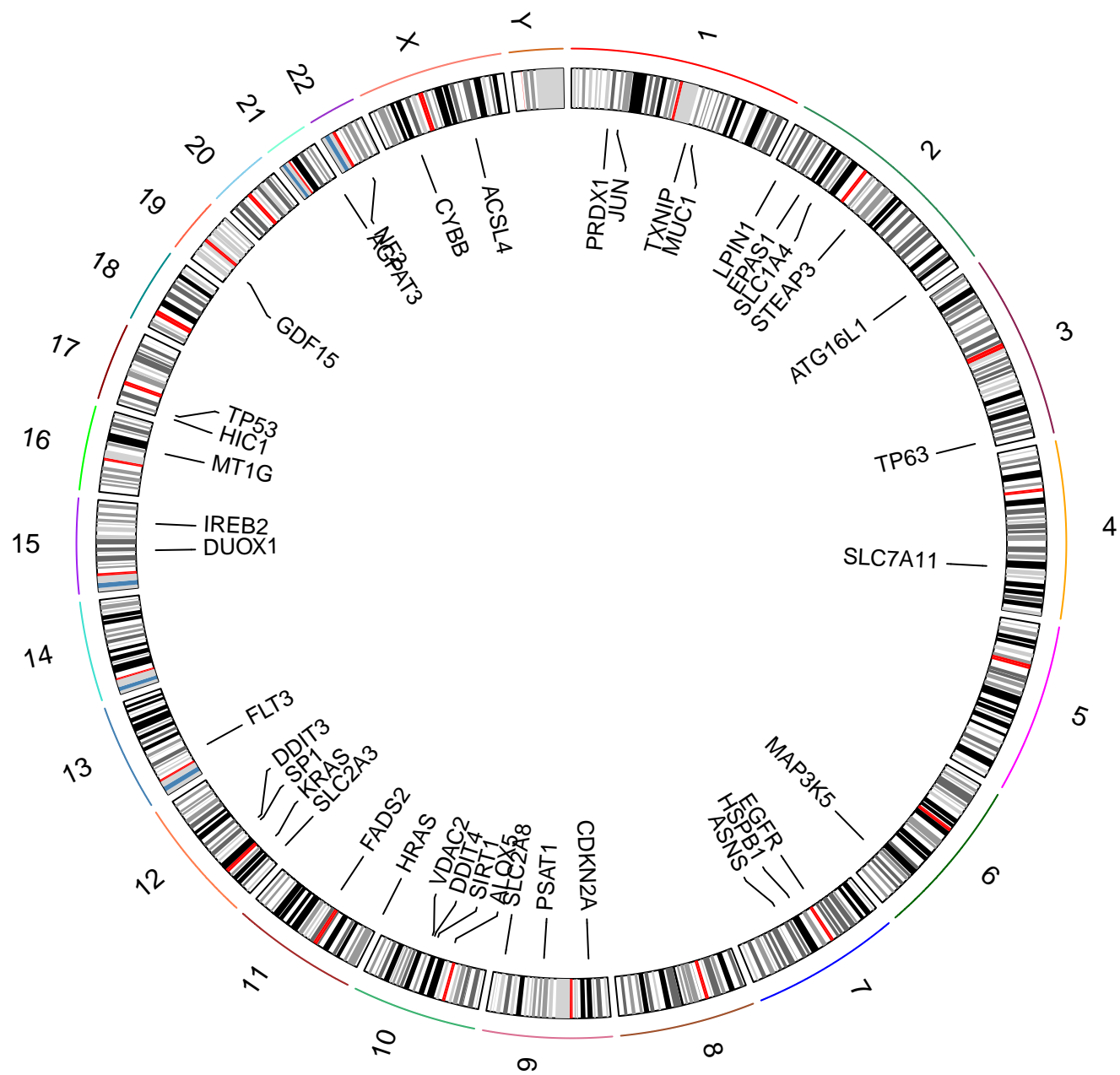

Supplement: Supplementary file 1 [file DataSheet_4.zip › Original data an R codes 1/10.Rcircos/RCircos.pdf]

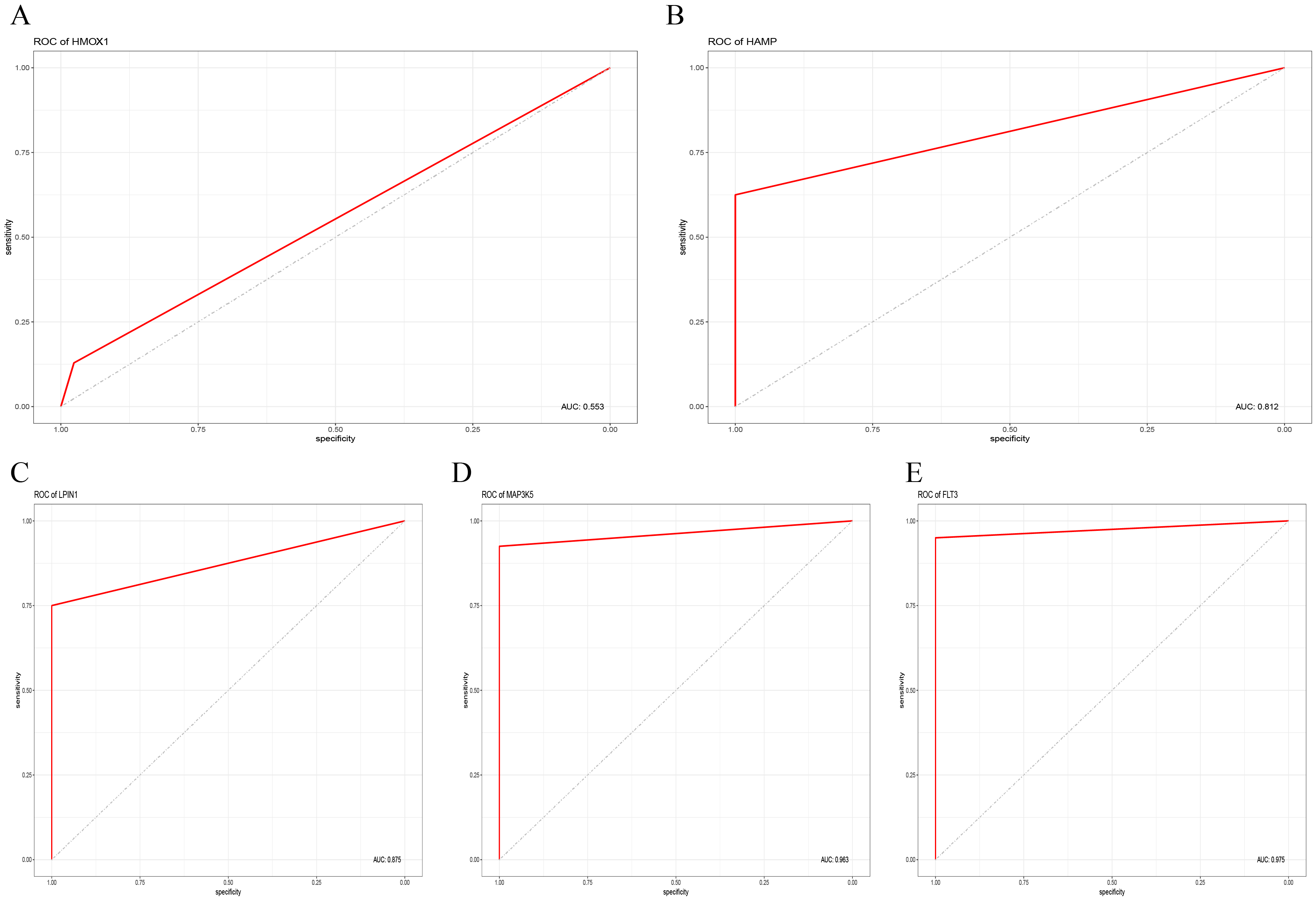

Supplement: Supplementary Figure 1 — ROC results for these 5 genes. (A-E) ROC results of HMOX1, HAMP, LPIN1, MAP3K5 and FLT3. [file Image_1.tif]
