# Supplementary material for: Identification of ferroptosis-associated biomarkers for the potential diagnosis and treatment of postmenopausal osteoporosis
Source: Front Endocrinol (Lausanne). 2022 Aug 29;13:986384. doi: 10.3389/fendo.2022.986384 (PMC9464919; doi:10.3389/fendo.2022.986384)
Supplement: Supplementary Table 1 — Identified 382 ferroptosis-associated genes. [file DataSheet_1.pdf]

SLC7A11
GPX4
AKR1C1
AKR1C2
AKR1C3
GPX4
RB1
HSPB1
HSF1
SLC7A11
GPX4
GCLC
SLC7A11
NFE2L2
SQSTM1
NQO1
HMOX1
FTH1
MUC1
SLC3A2
MT1G
NFE2L2
SLC40A1
SLC7A11
GPX4
SLC7A11
CISD1
SLC7A11
FANCD2
GPX4
NFE2L2
FTMT
HSPA5
ATF4
SLC7A11
GPX4
GPX4
HMOX1
ATF4
NFE2L2
TP53
SLC7A11
HELLS
SCD
FADS2
SRC
STAT3
NFE2L2
PML
MTOR
NFS1
TP63
SLC7A11
TP53
CDKN1A
MIR137
SLC40A1
GPX4

GPX4
ENPP2
VDAC2
FH
CISD2
SLC40A1
MIR9-1
MIR9-2
MIR9-3
CBS
NFE2L2
SQSTM1
GPX4
ISCU
FTH1
ACSL3
OTUB1
CD44
LINC00336
STAT3
BRD4
PRDX6
MIR17
SCD
SESN2
NF2
ARNTL
HIF1A
JUN
CA9
HSPA5
TMBIM4
HSPA5
PLIN2
MIR212
Fer1HCH
AIFM2
AIFM2
LAMP2
ZFP36
GPX4
PROM2
CHMP5
CHMP6
AKR1C1
AKR1C2
AKR1C3
CBS
NFE2L2
CAV1
GCH1
PTGS2
DUSP1
NOS2
NCF2
MT3
UBC
ALB

TXNRD1
SRXN1
GPX2
BNIP3
OXSR1
SELENOS
ANGPTL7
CHAC1
SLC7A11
DDIT4
LOC284561
ASNS
TSC22D3
DDIT3
JDP2
SESN2
SLC1A4
PCK2
TXNIP
VLDLR
GPT2
PSAT1
LURAP1L
SLC7A5
HERPUD1
XBP1
ATF3
SLC3A2
CBS
ATF4
ZNF419
KLHL24
TRIB3
ZFP69B
ATP6V1G2
VEGFA
GDF15
TUBE1
ARRDC3
CEBPG
SNORA16A
RGS4
BLOC1S5-TXNDC5
LOC390705
EIF2S1
KIM-1
IL6
CXCL2
RELA
HSD17B11
AGPAT3
SETD1B
HMOX1
TF
FTL
RPL8
ATP5MC3
TFRC

MAFG
IL33
FTH1
SLC40A1
TF
TFRC
FTH1
GPX4
HAMP
HSPB1
NFE2L2
STEAP3
DRD5
GPX4
DRD4
MAP3K5
MAPK14
SLC2A1
SLC2A3
SLC2A6
SLC2A8
SLC2A12
GLUT13
SLC2A14
EIF2AK4
EIF2S1
ATF4
ALOX5
ALOX12
ALOX15
ALOX5
ACSF2
IREB2
GPX4
HMGB1
HMOX1
NFE2L2
ELAVL1
SLC3A2
SLC7A11
TFAP2C
SP1
HBA1
NNMT
PLIN4
HIC1
STMN1
RRM2
CAPG
HNF4A
NGB
YWHAE
GABPB1
AURKA
MIR4715
RIPK1
PRDX1
MIR30B

RPL8
IREB2
ATP5MC3
CS
EMC2
ACSF2
NOX1
CYBB
NOX3
NOX4
NOX5
DUOX1
DUOX2
G6PD
PGD
VDAC2
PIK3CA
FLT3
SCP2
TP53
ACSL4
LPCAT3
NRAS
KRAS
HRAS
TF
TFRC
TFR2
SLC38A1
SLC1A5
GLS2
GOT1
CARS1
TP53
ALOX5
KEAP1
HMOX1
TP53
TP53
GLS2
ATG5
ATG7
NCOA4
TF
ALOX5
ALOX12
ALOX12B
ALOX15
ALOX15B
ALOXE3
PHKG2
TFRC
ACO1
IREB2
SLC38A1
GLS2
G6PDX
ULK1

ATG3
ATG4D
ATG5
BECN1
MAP1LC3A
GABARAPL2
GABARAPL1
ATG16L1
WIPI1
WIPI2
SNX4
ATG13
ULK2
NCOA4
ACSL4
TP53
SAT1
ALOX15
ACSL4
LPCAT3
ALOX15
ACSL4
KEAP1
EGFR
NOX4
MAPK3
MAPK1
BID
ACSL4
ZEB1
KEAP1
DPP4
ALOX15
ALOX12
CDKN2A
PEBP1
SOCS1
CDO1
MYB
HMOX1
MAPK8
MAPK9
MAPK1
MAPK3
SLC1A5
CHAC1
MAPK14
LINC00472
NOX4
GOT1
BECN1
PRKAA2
PRKAA1
ELAVL1
BAP1
TP53
ABCC1
ACSL4

MIR6852
ACVR1B
TGFBR1
BAP1
EPAS1
HILPDA
HIF1A
ALOX12
ACSL4
HMOX1
IFNG
ANO6
LPIN1
HMGB1
TNFAIP3
TLR4
NOX4
ATF3
ATM
YY1AP1
EGLN2
MIOX
TAZ
MTDH
IDH1
SIRT1
TAZ
BECN1
FBXW7
PANX1
DNAJB6
BACH1
ACSL4
LONP1
