# Supplementary material for: Identification of ferroptosis-associated biomarkers for the potential diagnosis and treatment of postmenopausal osteoporosis
Source: Front Endocrinol (Lausanne). 2022 Aug 29;13:986384. doi: 10.3389/fendo.2022.986384 (PMC9464919; doi:10.3389/fendo.2022.986384)
Supplement: Supplementary Table 2 — Acquired 45DEFAGs. [file DataSheet_2.pdf]

ID	GSM1369	GSM1369	GSM1369	GSM1369	GSM1369	GSM1369	GSM1369	GSM1369
HSPB1	6.47432	6.806613	6.778236	5.856816	6.726113	7.150083	6.833309	6.779139
SLC7A11	5.160335	5.220441	5.293512	5.198719	5.215008	5.183028	5.202907	5.256009
HMOX1	9.166371	8.858187	8.574116	8.902438	7.519898	8.818329	8.469485	8.65211
MUC1	6.231884	6.215888	6.21862	6.06252	6.352407	6.201466	6.209164	6.283929
MT1G	6.925139	7.163538	7.081477	7.091279	7.083227	7.164378	7.125402	6.9477
TP53	6.42182	7.660065	6.715644	6.972494	5.829142	6.993534	6.578648	7.070759
HELLS	5.40759	5.254531	5.32026	5.399093	5.699409	5.176458	5.228015	5.323259
FADS2	5.931621	6.15153	6.25603	6.064807	6.116777	6.060714	5.939339	6.798376
TP63	6.104809	5.924064	5.871618	5.953557	5.899582	5.908361	5.858449	6.032496
VDAC2	10.3512	10.46342	10.47515	10.44451	10.56939	10.3905	10.31125	10.24108
NF2	5.449423	5.639783	5.556439	5.532793	5.614974	5.575571	5.508703	5.585021
JUN	6.133227	6.061558	5.817451	5.770458	5.757707	5.644952	5.650311	5.752985
ZFP36	11.6114	11.6114	11.85371	11.62642	10.75184	11.49149	10.94077	11.68463
DDIT4	5.665481	6.167372	6.102238	6.059802	5.867763	5.947925	6.055779	5.820063
ASNS	5.491309	5.154868	5.307425	5.287477	5.589211	5.676249	5.304189	5.284554
DDIT3	7.690969	7.549541	7.800327	7.67899	7.704885	7.5821	7.519455	7.831332
SLC1A4	6.488889	6.7624	6.735699	6.792347	6.90465	6.608175	6.53459	6.722417
TXNIP	12.13536	12.39891	12.62801	12.54241	12.54241	12.12542	12.55524	12.22437
PSAT1	6.460188	6.516262	6.389802	6.593227	6.780446	6.469445	6.691558	6.728832
GDF15	5.344936	5.34205	5.47044	5.350551	5.372253	5.40268	5.367236	5.367544
AGPAT3	7.279343	7.691453	7.547698	7.417846	7.270087	7.500859	7.375125	7.51269
HAMP	5.658045	5.870151	5.715961	5.910595	6.794221	5.845062	5.842167	6.091285
STEAP3	7.182473	7.782676	7.130819	7.293097	7.213237	7.377762	7.4078	7.489807
MAP3K5	9.280482	8.639653	8.940294	9.039588	9.056143	8.850125	9.018748	8.686158
SLC2A3	8.919043	8.760293	8.888327	8.757096	8.470096	8.556812	8.46476	8.722101
SLC2A8	6.510164	6.551294	6.207956	6.197008	6.454956	6.344033	6.375738	6.587835
ALOX5	9.172906	8.764434	8.942783	8.889334	8.450012	9.053072	9.101186	9.084495
IREB2	5.555128	6.24874	6.120267	6.191851	6.632638	5.715461	5.934045	5.647937
SP1	6.930406	7.073138	7.023363	7.00834	6.485176	6.94911	6.727678	7.046528
HIC1	5.161746	5.072142	5.175747	5.195113	5.162109	5.171613	5.208185	5.116144
PRDX1	10.53116	10.26072	10.64433	10.70072	11.09152	10.18543	10.53116	10.89906
CYBB	9.381041	9.156633	9.319264	9.403644	9.062618	8.966081	9.398471	9.743632
DUOX1	5.599964	5.646744	5.709191	5.59925	5.665121	5.599425	5.687849	5.608456
FLT3	7.223959	7.264708	7.257333	7.163538	7.198851	7.330103	7.49803	7.071153
ACSL4	8.509607	8.555957	9.061718	8.990924	9.07137	8.373273	8.377847	8.677473
KRAS	8.077527	7.840127	8.263295	8.113821	8.533401	7.85436	8.089435	8.056456
HRAS	5.700962	6.137037	5.537771	5.443991	5.772163	5.617422	5.752222	5.846753
ALOX12B	5.620547	5.585273	5.429179	5.335873	5.235262	5.590225	5.389165	5.578831
ALOXE3	6.056017	5.977214	6.004586	6.051319	6.246235	6.015797	6.266059	6.144415
ATG16L1	5.743124	5.283588	5.293142	5.358198	5.602286	5.328439	5.390455	5.45719
EGFR	6.468967	6.568508	6.602598	6.564398	6.524028	6.476764	6.55598	6.598568
CDKN2A	5.770931	5.813711	5.693889	5.734001	5.744342	5.841131	5.805658	5.910595
EPAS1	7.121738	6.982023	6.933323	6.996796	6.926246	7.06104	6.931764	6.939608
LPIN1	6.569958	6.328728	6.40639	6.347709	6.38951	6.543956	6.505338	6.419759
SIRT1	7.847366	7.405502	8.224838	8.231806	8.141383	7.971851	7.840127	7.714992

GSM1369	GSM1369	GSM1369	GSM1369	GSM1369	GSM1369	GSM1369	GSM1369	GSM1369
7.658587	6.383536	6.782631	6.4414	7.668324	6.637935	7.179495	7.203002	7.354038
5.286378	5.212501	5.159715	5.265026	5.150404	5.116894	5.249969	5.276464	5.16834
8.726871	8.595273	8.778611	8.478124	9.171823	8.927769	9.058883	8.940294	9.529178
6.285271	6.241461	6.379401	6.326152	6.276416	6.347709	6.191512	6.2918	6.324963
7.059982	7.128835	7.236406	7.343493	7.145984	7.110527	6.960487	7.208328	7.201143
6.700962	6.50099	6.700329	6.854403	7.065693	7.223244	6.434731	6.885271	6.942203
5.431439	5.157392	5.439072	5.262147	5.289287	5.403581	5.34936	5.45719	5.304452
6.187258	6.369257	5.941483	6.075784	6.55892	5.933328	6.439215	6.239097	6.607848
5.966035	5.953793	5.952114	5.985674	5.907149	5.98276	5.908001	5.913901	5.957135
10.43072	10.48644	10.66667	10.52307	10.48951	10.60969	10.4392	10.38774	10.47821
5.562834	5.515208	5.573643	5.540336	5.627432	5.624718	5.61554	5.519145	5.582327
5.613954	5.636903	5.74144	5.617999	5.581838	5.531255	5.669956	5.677975	5.628161
11.1358	11.28208	11.26287	10.68651	10.81131	10.845	10.95288	10.81131	11.21613
5.864971	5.812912	5.88599	5.758466	5.976494	5.912032	6.327109	5.857518	6.15297
5.170305	5.410831	5.543335	5.334592	5.396857	5.179416	5.414404	5.449941	5.395686
7.666361	8.003186	7.921559	7.694464	7.883429	7.681543	7.586678	7.548573	8.130707
6.563584	6.881047	6.75017	6.813377	6.803109	6.883381	6.744723	6.87678	6.858725
12.60434	12.39891	12.62801	12.64024	12.32351	12.28035	12.31064	12.28035	12.17813
6.574404	6.45053	6.67187	6.59524	6.534864	6.462884	6.63165	6.534486	6.743556
5.429746	5.34412	5.443681	5.322394	5.440571	5.419863	5.352673	5.524257	5.427543
7.504863	7.395854	7.177552	7.303717	7.561199	7.403344	7.247436	7.270455	7.773611
6.161369	5.660608	5.726748	5.692963	6.343729	5.795604	6.136007	5.746432	6.610761
7.0126	7.268913	7.131426	7.391786	7.741303	7.109363	7.014812	7.35235	7.287828
8.889334	9.162899	9.112261	9.049333	8.399154	8.939489	9.165116	8.649031	8.624189
8.865333	8.565163	8.415192	8.381385	8.297196	8.642879	8.43567	7.982381	8.047281
6.434994	6.365515	6.362177	6.260818	6.759999	6.563584	6.520596	6.241225	6.652607
8.954053	8.836349	8.682978	8.769756	9.262762	8.852549	8.810373	9.198874	9.374366
5.770458	5.761832	6.23406	5.728742	5.810491	5.903794	6.203418	5.735163	5.596571
7.351932	6.578516	6.793883	6.980698	6.693784	6.899629	6.73221	7.064163	7.21033
5.269359	5.274295	5.253285	5.121668	5.404132	5.235551	5.19776	5.514114	5.549115
10.8528	10.49833	10.85763	10.68013	10.39953	10.92398	10.73748	10.845	10.48644
9.718125	8.717989	9.66966	9.44757	9.242277	9.53609	9.282446	8.672993	9.418538
5.628161	5.596822	5.695683	5.638322	5.655405	5.576627	5.610525	5.511266	5.73706
7.234225	7.043274	7.068665	7.116826	7.400796	6.987784	7.83742	6.972494	7.352775
9.102093	8.842229	9.015946	8.567477	8.446544	9.007194	9.063469	8.214437	8.330641
8.066525	8.265332	8.181429	8.205786	7.965559	8.224838	8.25549	8.163682	8.003825
5.869671	5.592258	5.409304	5.451651	5.739607	5.672419	5.776198	5.713025	6.037376
5.583654	5.770235	5.684162	5.528105	5.67123	5.547133	5.384824	5.585273	5.848791
6.163517	5.908921	5.980642	6.2523	6.099234	5.877022	6.229606	6.018402	6.063794
5.486877	5.376519	5.366986	5.469553	5.439409	5.545119	5.531506	5.329033	5.386925
6.474597	6.578904	6.58118	6.628288	6.578648	6.557715	6.466626	6.460473	6.541102
5.837273	5.747131	5.862027	5.991528	5.922073	5.731067	5.883751	5.847313	5.91949
6.968389	6.955222	6.877376	7.020751	7.020034	6.82362	6.978328	7.10094	6.88589
6.368043	6.478327	6.440284	6.359855	6.556621	6.465509	6.612897	6.583249	6.476516
7.953775	8.150663	8.26983	7.932724	7.552802	8.140626	7.912336	7.813269	7.329677

GSM1369	GSM1369	GSM1369	GSM1369	GSM1369	GSM1369	GSM1369	GSM1369	GSM1369
7.365807	6.300888	7.981095	7.769282	6.985309	6.79909	7.03477	7.357385	7.060686
5.246984	5.170305	5.234982	5.233033	5.266698	5.217562	5.215365	5.187193	5.23199
8.941046	8.710449	8.832303	9.329995	8.797729	9.013074	9.118273	8.851888	8.5856
6.098518	6.253026	6.059519	6.204636	6.257567	6.162281	6.217886	6.199284	6.214578
7.089734	6.917133	6.901302	7.103811	7.11947	7.216464	7.050227	7.233113	7.208721
7.163538	6.669903	6.671193	7.235817	6.602598	6.486316	6.481606	6.96931	6.505871
5.286127	5.29057	5.505527	5.233336	5.171928	5.229073	5.295377	5.298406	5.315225
6.31628	6.313194	6.549895	6.242713	6.32736	6.495004	6.263433	6.216928	6.315792
5.905482	5.861768	6.032965	5.928247	5.876343	5.887307	5.905273	5.917835	5.962146
10.37473	10.62577	10.44451	10.34096	10.48366	10.26801	10.49281	10.17659	10.58873
5.728462	5.526429	5.614699	5.627188	5.548157	5.630748	5.586662	5.611289	5.565926
5.789487	5.869155	5.612753	5.649606	5.54533	5.559544	5.698713	5.576333	5.628478
11.52509	11.12094	11.22563	11.03154	11.12954	10.67675	10.36921	11.11124	10.59176
5.961691	6.171811	5.937329	6.46629	5.900548	6.065037	6.071602	5.890946	6.047494
5.281704	5.419863	5.307425	5.316623	5.32131	5.347799	5.389165	5.516922	5.26188
7.611245	7.785758	7.518028	7.543594	7.535413	7.551394	7.391429	7.644455	7.693364
6.665894	6.775565	6.530651	6.618488	6.760269	6.581751	6.696612	6.585458	6.693473
12.09203	12.50103	12.33784	12.44677	12.31064	12.62801	12.69235	12.51559	12.37008
6.542221	6.270885	6.510474	6.504811	6.528256	6.562994	6.46765	6.46418	6.524028
5.268629	5.443095	5.445369	5.427267	5.375233	5.499341	5.4822	5.377039	5.411854
7.360696	7.191222	7.445822	7.426896	7.580283	7.122294	7.261338	7.493794	7.532331
6.447115	5.561148	6.351166	6.046166	5.900318	6.039702	5.890484	5.818232	5.840661
7.447753	7.326133	7.321451	7.430494	7.088709	7.021312	7.169248	7.090125	7.181766
8.930824	8.785399	8.712498	8.860107	8.575365	8.918074	9.067166	8.961474	8.553705
8.687768	8.49385	8.713213	8.153697	8.375813	8.760293	8.889334	8.663014	9.324937
6.474597	6.395842	6.515746	6.470302	6.412539	6.445407	6.403853	6.372917	6.483813
8.869407	8.789082	9.048213	9.001404	9.042857	9.107743	8.902438	9.040689	8.961474
5.817451	6.319111	5.760111	5.890025	6.032275	5.766204	6.025433	5.885417	5.927488
6.387683	6.778837	6.963322	6.911037	7.128835	7.124254	6.918416	6.964798	6.748948
5.138691	5.517056	5.307147	5.284242	5.253912	5.308257	5.224988	5.230705	5.188107
10.27774	10.86981	11.03552	10.44146	10.97423	11.03969	10.74121	10.76942	11.00327
9.212307	9.407309	9.658677	9.246595	9.040689	9.293071	9.181538	9.316061	9.726526
5.624718	5.720504	5.629871	5.626942	5.607931	5.632899	5.586379	5.586662	5.647937
7.31699	7.182086	7.482469	7.172017	6.978328	7.010075	7.247436	7.133015	6.970094
8.273299	8.545395	8.716382	8.645213	8.43701	8.501349	8.973988	8.573034	8.388893
7.982871	8.222942	7.922034	7.979477	8.060126	8.158714	8.220268	8.012886	8.00721
6.055517	5.450575	5.685619	5.87059	5.752432	5.634119	5.646218	5.711159	5.749714
5.558225	5.621912	5.67123	5.499899	5.509274	5.511883	5.630965	5.543335	5.510966
6.036449	5.80548	6.099234	6.101486	5.947925	6.153921	6.037642	6.035456	6.033524
5.44228	5.498533	5.513306	5.383575	5.228743	5.469003	5.449941	5.498533	5.3545
6.510164	6.399721	6.343291	6.550743	6.478327	6.466891	6.44452	6.461453	6.51719
5.821271	5.788108	5.755975	5.799377	5.812397	5.853872	5.710211	5.852206	5.864722
6.951933	7.331588	6.886237	6.922456	6.873911	7.221602	6.942203	6.981046	6.917432
6.572193	6.413072	6.458355	6.578384	6.536943	6.560368	6.549895	6.556296	6.511889
7.697474	8.179092	8.021693	7.758683	8.051305	8.015896	8.306974	7.527661	7.46745

GSM1369	GSM1369	GSM1369	GSM1369	GSM1369	GSM1369	GSM1369	GSM1369	GSM1369
6.696952	6.91402	6.824773	6.84002	6.869195	8.008798	6.84777	7.366195	6.773183
5.256308	5.283924	5.195461	5.15303	5.192856	5.110601	5.181571	5.19776	5.20461
8.897612	8.690603	8.494878	8.941046	8.848456	9.116241	9.141065	9.172906	9.512657
6.194352	6.204398	6.262739	6.352407	6.286283	6.220805	6.305811	6.114082	6.377313
7.111321	7.145211	7.123842	7.121738	6.994607	7.090125	6.93931	7.273272	7.227393
6.72276	6.769045	6.869195	6.632504	6.688335	7.008701	7.203002	6.955222	7.275583
5.094732	5.343025	5.20606	5.381172	5.371421	5.32026	5.194967	5.281121	5.330599
6.021945	6.086025	6.304197	6.257838	6.178428	6.146158	6.826806	6.369494	6.257344
5.883029	5.955676	5.997173	5.933328	5.964588	5.979177	6.013348	5.928039	5.932319
10.30147	10.46921	10.07068	10.33	10.49281	10.45294	10.24794	10.45799	10.23848
5.531781	5.543845	5.596822	5.576627	5.617187	5.67123	5.634682	5.647713	5.61554
5.591498	5.718338	5.670867	5.613954	5.859918	5.74121	5.704768	5.774377	5.586379
10.5568	11.61825	11.03154	11.18589	11.89889	11.74976	10.97423	11.57197	10.85763
5.97677	6.092989	6.117232	5.944753	5.995605	6.125768	6.387683	6.033524	6.146414
5.339264	5.205651	5.455521	5.574762	5.254209	5.403581	5.428081	5.429892	5.387166
7.564498	7.786759	8.034413	7.776937	7.918176	7.919441	7.891359	7.891359	8.056456
6.532429	6.683597	6.678194	6.803389	6.55917	6.74445	6.792197	6.685713	6.601159
12.56607	12.33784	12.55524	12.51559	12.1677	12.2125	12.32351	12.00752	12.73377
6.589841	6.596163	6.391418	6.495128	6.460473	6.559821	6.638479	6.500014	6.480015
5.528618	5.462391	5.547264	5.369542	5.373439	5.491789	5.419609	5.447054	5.425566
7.46745	7.428607	7.484338	7.385224	7.752609	7.434364	7.15534	7.600319	7.358995
5.770235	5.921521	5.757945	6.30961	6.78753	6.233312	6.33027	6.077915	6.126478
7.317787	7.229722	7.522291	7.372629	7.2666	7.364537	7.371473	7.139012	7.287434
8.951075	8.866027	8.608776	8.902438	8.814099	8.851888	8.789082	8.698543	8.757874
8.226104	8.369565	8.085312	8.387508	9.377129	8.583166	8.15802	7.988062	8.224838
6.464654	6.539775	6.609951	6.539174	6.723021	6.579811	6.417972	6.675249	6.657004
8.919822	9.022974	8.99262	9.16865	9.125525	9.092479	9.065342	9.157674	9.155728
5.843918	5.83569	5.775269	5.73033	5.618783	5.838449	5.824455	5.413827	6.024272
6.865252	7.128145	6.723661	7.124254	7.046155	7.204505	7.279343	6.88499	7.159799
5.318469	5.142969	5.303574	5.351784	5.296891	5.471574	5.349066	5.401214	5.538428
10.72424	10.81131	10.845	10.72424	10.60669	10.59775	10.64433	10.60383	10.62941
9.442984	9.476397	9.234526	9.420294	9.426826	9.151824	9.402349	9.152807	9.375819
5.664412	5.662121	5.640022	5.691731	5.676249	5.70684	5.710674	5.598534	5.660059
7.556482	7.325118	7.129673	7.343882	7.463481	7.258469	7.282247	7.313762	7.221183
8.516531	8.692871	7.886328	8.154901	8.605611	7.87201	8.372595	7.782183	8.423099
7.994468	8.056456	7.903878	7.83742	7.969743	7.802361	7.714992	7.346358	7.692855
5.76484	5.821011	5.806794	6.220805	6.022753	6.093466	5.988141	5.973195	6.026936
5.515622	5.537338	5.658614	5.543572	5.597574	5.648887	5.497775	5.622033	5.581051
5.96579	6.109301	6.276961	6.071602	6.082183	5.966504	6.205935	6.012101	6.231614
5.301669	5.308882	5.375233	5.458248	5.371719	5.566443	5.280214	5.431762	5.563091
6.416441	6.56325	6.695682	6.593227	6.625744	6.562435	6.489419	6.534382	6.582324
5.823538	5.774112	5.915127	5.917584	5.887033	5.919763	5.8187	5.784907	5.931621
6.916821	6.82575	6.788136	6.965099	6.932087	6.99885	6.82575	7.106508	6.862338
6.510749	6.597922	6.728431	6.516901	6.387444	6.594585	6.382241	6.616061	6.478666
8.102567	8.010027	7.786265	7.389749	7.848599	7.597105	7.260896	7.557011	6.759999

GSM1369	GSM1369	GSM1369	GSM1369	GSM1369	GSM1369	GSM1369	GSM1369	GSM1369
7.148207	6.251258	6.874615	7.200002	7.176823	6.608466	7.31174	6.149288	5.960942
5.229367	5.326926	5.232282	5.243453	5.176458	5.283924	5.327209	5.203876	5.19571
8.487627	8.264611	9.053994	8.713213	8.619457	8.101241	7.628769	8.110699	8.583825
6.247	6.297761	6.291571	6.256643	6.243186	6.332974	6.303651	6.389036	6.330005
7.255825	7.140894	7.226603	7.039431	7.170436	7.050227	6.962723	7.135895	7.142816
6.76835	6.642771	6.773183	7.20333	7.212854	6.577752	6.1136	6.040901	7.16097
5.468047	5.473486	5.165875	5.276706	5.313332	5.457481	5.372775	5.152313	5.372381
6.493134	6.171084	6.21335	6.287246	6.794559	6.320134	6.035671	5.868665	5.878057
5.899049	5.878536	5.856579	5.979177	6.001099	5.947058	6.006506	5.895163	5.97509
10.58873	10.62256	10.3137	10.52845	10.49833	10.65981	10.46342	10.40221	10.39953
5.553621	5.564137	5.553351	5.634682	5.609248	5.473798	5.659391	5.522951	5.54622
6.025955	5.466046	5.843379	5.587708	5.657563	5.566922	5.69315	5.431762	5.595692
11.82542	10.52562	12.23932	11.22107	11.6114	10.3137	10.70072	10.53116	11.26287
6.054992	6.023062	6.097831	6.216167	6.190812	6.24874	5.980642	5.857311	6.012576
6.608175	5.379715	5.124449	5.362047	5.570822	6.429715	5.341686	5.589211	5.25084
8.063459	7.785758	8.000391	7.992013	7.942198	7.995032	7.571225	8.015896	7.941525
7.310961	6.796198	6.771858	6.717554	6.743556	6.922123	6.564132	6.390121	6.780124
11.69874	12.56607	12.31064	12.43078	12.52826	12.45892	12.73377	12.64024	12.43078
6.890214	6.604608	6.515996	6.311637	6.579471	6.573596	6.718966	6.425674	6.44926
5.494453	5.455244	5.459005	5.535208	5.39453	5.358762	5.389431	5.453862	5.407353
7.766111	7.204505	7.398179	7.444561	7.66106	6.995011	7.341336	6.728587	7.494731
5.918742	5.574762	6.047494	5.904783	5.903794	5.66661	6.021694	5.5831	5.766537
7.04953	7.43313	7.426478	7.221976	7.354946	7.148207	7.358226	7.647409	7.325118
8.194613	8.963404	8.90513	8.579268	8.480261	8.426433	9.001404	8.873138	8.712498
8.479618	8.258476	8.499971	7.771402	8.261227	7.874245	9.101186	8.058335	8.052559
6.584885	6.374757	6.423699	6.590577	6.440868	6.376841	6.42342	6.372917	6.35526
8.827213	8.87131	9.07137	9.244458	8.926064	8.737685	8.558284	8.52742	8.966081
5.377346	5.854395	5.661373	5.935785	5.63732	5.646485	6.095181	5.511883	5.856579
7.062059	6.919532	7.01518	7.027335	7.042174	6.84777	6.949824	6.828655	6.911037
5.312695	5.194491	5.322394	5.305976	5.194145	5.301354	5.020603	5.489812	5.40814
10.91572	10.80782	10.82196	10.62941	10.29395	11.15735	10.97815	10.91197	10.69347
9.221152	9.287031	9.534621	9.221152	9.152807	9.187826	9.360843	9.223396	9.431077
5.647199	5.650067	5.716953	5.61926	5.691249	5.587487	5.640497	5.776485	5.608742
7.284769	7.714026	7.070359	7.129673	7.527661	7.44243	7.461147	7.278668	7.470818
7.949692	8.733745	8.272648	8.452343	8.404925	8.408645	8.726051	8.44579	8.38957
7.859489	7.869025	8.015396	7.9085	7.904342	8.042759	8.474355	8.046257	7.990969
5.874725	5.74832	5.827256	5.790852	5.850547	5.559273	5.723845	5.419736	5.993823
5.817929	5.49093	5.733037	5.484694	5.580464	5.505147	5.297645	5.680544	5.602819
5.985437	6.058531	6.202648	6.128491	6.198768	6.107547	6.294207	6.063056	6.075565
5.536492	5.365078	5.600221	5.400948	5.501557	5.389792	5.383905	5.388524	5.412138
6.612897	6.543667	6.609327	6.601692	6.632772	6.527689	6.49056	6.592934	6.586375
5.927212	5.88035	5.821011	5.886114	5.908455	5.931408	5.874967	5.924064	5.870876
6.888769	7.060686	6.90198	6.967088	6.830116	7.041853	6.850972	7.140591	7.085803
6.541666	6.611062	6.49056	6.535879	6.466626	6.717554	6.567252	6.676042	6.593998
7.194216	7.724341	7.352775	7.533214	7.358226	7.665792	7.723307	7.339752	7.658128

GSM1369	GSM1369	GSM1369	GSM1369	GSM1369	GSM1369	GSM1369	GSM1369	GSM1369
6.22859	7.156108	6.514348	6.476516	6.188389	6.219569	6.466015	6.607356	7.233845
5.227692	5.19194	5.244067	5.200678	5.268293	5.260693	5.256978	5.305976	5.223847
8.291393	9.229147	8.407864	8.425061	8.854491	8.552037	8.917179	8.968102	9.060697
6.242262	6.258359	6.354202	6.236244	6.384547	6.179248	6.293036	6.258567	6.210904
7.079166	7.06104	7.060686	7.221602	7.127379	7.203757	7.243718	7.157572	7.13221
6.530155	6.658832	6.607848	6.388265	6.333241	6.487489	7.153376	6.470739	6.70506
5.234316	5.350551	5.225758	5.327209	5.109794	5.157392	5.317486	5.12926	5.213446
5.93187	6.212311	6.032965	6.22695	6.618778	6.335693	6.08289	6.106211	6.273898
5.881776	5.954831	5.989687	5.920768	6.05649	6.103791	5.952114	6.000351	5.917158
10.59775	10.62256	10.77731	10.46128	10.6003	10.55104	10.49549	10.67318	10.52845
5.481292	5.623166	5.528105	5.537898	5.528909	5.532542	5.585557	5.414972	5.624485
5.650778	5.735163	5.507126	5.56938	5.471574	5.447054	5.580191	5.54533	5.742647
11.15735	11.35901	10.33279	10.45578	10.4334	10.76174	11.1635	11.02286	11.23103
6.169227	6.244321	6.094673	5.996645	5.731824	5.789487	6.073503	5.8766	6.108318
5.777218	5.408945	5.491159	5.795604	5.434613	5.693889	5.396291	5.723845	5.372005
7.66522	7.950145	7.772061	7.914937	7.786759	7.893636	7.796087	7.856608	7.928857
6.785046	6.628012	6.483533	6.587045	6.62685	6.762774	6.820495	6.688024	6.619334
12.58019	12.38507	12.59131	12.60434	12.54241	12.50103	12.13536	12.69235	12.25213
6.508748	6.452371	6.607356	6.40639	6.470887	6.310558	6.467421	6.556889	6.656676
5.40574	5.532028	5.433164	5.361136	5.596571	5.516027	5.551111	5.560914	5.603094
7.374664	6.987364	7.264349	7.282247	7.130819	7.323342	7.43313	7.254272	6.994607
5.752222	6.265417	5.851442	5.312355	5.694362	5.74761	5.7181	5.707538	6.401665
7.120198	7.467826	7.068665	7.529985	7.216125	7.49714	7.733901	7.084633	7.153202
8.681027	8.674874	8.847584	8.706187	8.748601	8.725116	8.534781	8.999316	8.534167
8.333244	8.312116	8.281548	8.247842	8.174599	8.345138	8.265332	8.503431	7.758181
6.405602	6.52034	6.242449	6.41335	6.458486	6.330005	6.482414	6.307335	6.505871
8.739955	8.981496	8.701022	8.83722	8.947287	8.921753	8.919822	8.82269	8.867641
5.712128	6.053553	5.714964	5.714964	5.638322	5.745964	5.705832	5.466872	5.698461
6.971113	6.930027	6.96546	6.590893	6.295474	6.837694	6.746979	7.110527	6.397079
5.301937	5.345214	5.294148	5.317373	5.390724	5.345802	5.301669	5.236596	5.399478
11.0809	10.81131	10.83636	10.97815	10.61945	10.91967	10.4334	10.76549	11.06662
9.431077	9.354926	9.787263	9.299831	9.350075	9.469055	9.472062	9.425517	9.028874
5.497213	5.720504	5.606892	5.596571	5.591765	5.657313	5.568656	5.6137	5.592515
7.373118	7.350778	7.524805	7.870116	7.498541	7.329677	7.309775	7.687896	7.247436
8.450012	8.483136	8.358372	8.53173	8.560113	8.280672	8.295113	8.471543	8.505637
7.95931	7.799716	7.788164	7.897059	7.850692	8.044013	7.786759	8.13952	7.992013
5.51878	5.906897	5.358198	5.528358	5.616384	5.607151	5.715705	5.58445	5.980511
5.543011	5.837053	5.853872	5.757151	5.600944	5.988944	5.667639	5.688316	5.785419
6.146655	6.109301	6.063564	6.158495	6.204148	6.137303	6.113328	6.058294	5.99284
5.535455	5.338421	5.288981	5.396291	5.391007	5.368961	5.413961	5.419609	5.426477
6.537792	6.657343	6.535646	6.574688	6.664129	6.616619	6.667746	6.525121	6.580577
5.731322	5.829142	5.854877	5.937587	5.906897	6.032749	5.913368	5.766204	5.777971
6.901302	6.884677	7.124254	7.066085	6.967759	7.050899	6.965099	6.935112	7.002469
6.67285	6.40121	6.60231	6.610761	6.793593	6.611966	6.47247	6.590893	6.40325
7.73269	7.48534	7.913624	7.5995	7.529985	7.488416	7.471196	8.011746	7.719592

GSM1369(	GSM1369(	GSM1369(	GSM1369(	GSM1369(	GSM1369(	GSM1369(	GSM1369(	GSM1369(
6.933879	6.803109	6.523719	6.727415	6.749665	6.441151	7.475299	6.052225	6.471891
5.2487	5.183675	5.252565	5.226084	5.278289	5.323821	5.307754	5.247286	5.32528
8.910609	8.456212	8.581593	8.241933	8.953103	8.434837	9.021931	8.249884	8.247842
6.129187	6.317269	6.279596	6.175561	6.334443	6.254525	6.239348	6.288884	6.195528
7.118314	7.05787	7.124614	7.140591	7.230108	7.051575	7.260896	7.246336	7.274851
7.354946	6.613532	6.493657	6.372917	6.772181	6.888128	7.611245	6.373933	6.586693
5.322703	5.250558	5.376519	5.125083	5.212814	5.330264	5.098595	5.426477	5.166602
6.32241	6.011514	5.956741	6.163011	6.222294	5.980143	6.224997	5.96579	6.076044
5.996135	5.93711	5.955131	5.996135	5.920768	5.982029	6.036674	5.945609	5.929701
10.49281	10.50801	10.82196	10.56264	10.70721	10.68013	10.41386	10.60383	10.48951
5.493096	5.503635	5.550867	5.490383	5.519716	5.519145	5.540049	5.484418	5.54622
5.547877	5.564653	5.633137	5.686807	5.641234	5.532793	5.5221	5.634389	5.64221
11.22563	10.60669	10.86981	10.88185	10.89026	10.36159	10.94886	10.90323	11.15735
5.86953	6.226211	5.97697	6.131963	6.387993	6.647851	6.406889	6.185388	6.221474
5.357937	5.521464	5.663876	5.384554	5.441437	5.495879	5.678691	5.459819	5.506063
7.887362	7.687896	7.441028	7.776163	7.950145	7.794432	7.89825	7.770349	7.896504
6.810825	6.680472	6.610476	6.639429	6.663306	6.59915	6.986041	6.630267	6.528256
12.47324	12.62801	12.61705	12.64024	12.67814	12.78567	12.65114	12.41556	12.54241
6.471624	6.531813	6.010374	6.304529	6.64076	6.5664	6.44999	6.543667	6.437561
5.395075	5.433164	5.600655	5.455244	5.458401	5.456578	5.505003	5.557225	5.519145
7.435091	7.313325	7.231605	7.423886	7.33481	7.365807	7.57953	7.688464	7.280614
5.628796	5.697287	5.708735	5.606117	5.955932	5.705004	5.612753	5.679447	5.669956
7.236623	7.333959	7.433973	7.397195	7.429548	7.396325	7.341336	7.380743	7.453075
8.82884	8.6023	8.619457	8.838824	8.877119	8.823548	8.667101	8.659228	8.529608
8.268655	8.347269	8.279404	8.537848	8.340128	8.540316	7.885758	8.486191	8.316749
6.426505	6.401946	6.287506	6.274647	6.411452	6.307335	6.408122	6.394497	6.50154
9.005119	8.879159	8.686158	8.672993	8.715565	8.944511	8.981496	8.952025	8.861852
5.626008	5.478582	5.654882	5.682706	5.516193	5.784907	5.710211	5.746432	5.547264
6.636671	7.01518	7.233845	6.852035	6.738354	6.821497	6.883075	6.793593	6.769893
5.177745	5.380578	5.523476	5.332928	5.224818	5.133845	5.223847	5.34412	5.566701
10.53349	11.04426	10.66323	10.96554	10.58215	10.46342	10.67318	10.96972	10.71453
9.440376	9.544574	9.718125	9.476397	9.44757	9.458265	9.317113	9.581979	9.395846
5.716234	5.679447	5.619756	5.62108	5.535976	5.478885	5.563914	5.543845	5.497482
7.408849	7.457043	7.042552	7.601343	7.643207	8.002612	6.968652	7.719021	7.186733
8.385663	8.578556	8.211962	8.555154	8.362788	8.434187	8.42574	8.339463	8.405696
7.950145	8.027423	8.044596	8.06851	8.199802	8.148633	8.058335	8.060126	7.913624
5.703784	5.65659	5.612753	5.497775	5.611046	5.555975	5.70451	5.755524	5.610779
5.65383	5.640727	5.723322	5.612303	5.637595	5.613196	5.74121	5.624485	5.803488
6.213963	6.013112	5.977752	6.107547	6.264138	6.195045	6.012808	6.19243	6.178521
5.246675	5.224818	5.411348	5.36482	5.223847	5.37997	5.299385	5.273033	5.430038
6.67024	6.605958	6.582324	6.583249	6.500014	6.576519	6.578904	6.560368	6.662036
5.79764	5.877501	5.981789	5.901635	5.838223	5.941238	5.913474	5.871369	5.950054
7.081113	6.837988	7.130819	7.030743	6.984301	7.07003	7.081113	7.125402	6.951313
6.582066	6.568765	6.704108	6.80891	6.706063	6.686329	6.529111	6.598873	6.538331
7.752609	7.727081	7.831951	7.753901	7.965559	7.953142	7.468287	7.926166	7.661594

GSM1369(	GSM1369(	GSM1369(	GSM1369(	GSM1369(	GSM1369(	GSM1369(	GSM1369(	GSM1369(
6.190106	7.310216	7.172809	6.736007	6.147146	6.37633	6.196754	6.05649	6.523149
5.318191	5.234627	5.304783	5.090554	5.250558	5.269026	5.227692	5.243103	5.228015
8.511137	8.110034	8.882814	8.89132	8.635606	8.286714	8.611672	7.976625	8.199802
6.261745	6.42796	6.347922	6.214802	6.281768	6.355005	6.214802	6.366495	6.275146
7.278668	7.104574	7.24795	7.29862	7.238062	7.125402	7.205504	7.254272	7.187482
6.335432	6.769596	6.770204	6.85301	7.06104	6.729517	7.340167	6.661524	6.5149
5.18487	5.209589	5.284858	5.239751	5.13192	5.298114	5.273033	5.305708	5.203547
6.137796	6.389036	6.084111	6.200027	6.020102	6.099452	6.302838	6.132249	6.416998
5.91536	5.985437	6.027228	6.010628	6.067391	5.967735	6.021194	5.958137	5.955932
10.78819	10.64692	10.50801	10.19753	10.24318	10.41704	10.40221	10.58215	10.54816
5.498029	5.533323	5.505003	5.543572	5.558225	5.539512	5.54622	5.568923	5.573883
5.539248	5.524506	5.429469	5.706354	5.521849	5.496939	5.407118	5.534966	5.630965
10.53116	10.5113	10.79625	11.40531	10.97423	10.49549	10.91197	10.89906	10.83636
6.156942	6.078379	6.095654	6.150059	6.411167	5.855622	6.216167	6.085767	6.175812
5.472704	5.564369	5.486033	5.713025	5.484845	5.509554	5.396527	5.360521	5.469876
7.822543	7.943257	8.161272	7.976625	8.142652	7.817643	7.768712	7.8463	8.307679
6.551817	6.637576	6.922123	6.511325	6.615521	6.720876	6.687659	6.662358	6.58406
12.66527	12.48865	12.74772	12.44677	12.1677	12.74772	12.25213	12.64024	12.54241
6.373656	6.301779	6.637935	6.545864	6.459663	6.452626	6.314988	6.23706	6.328464
5.598107	5.563091	5.3545	5.446715	5.517725	5.402962	5.597073	5.384554	5.435659
7.216464	7.327187	7.59324	7.142407	7.302037	7.305789	7.229722	7.085351	7.319027
5.42133	5.762094	5.647445	5.56938	5.510743	5.579092	5.751413	5.685619	5.867763
7.089096	7.483185	7.224466	7.558823	7.835095	7.46962	7.6359	7.568745	7.489338
8.785399	8.743116	8.645935	8.434187	8.33133	8.617138	8.586583	8.889334	8.739295
8.322996	8.206521	7.849626	7.95437	8.247132	8.038554	8.573034	8.101885	7.845756
6.451866	6.323182	6.499148	6.545048	6.428294	6.49471	6.4107	6.357734	6.52712
8.796137	8.894783	9.098178	9.302114	8.882814	8.791696	9.116241	8.861056	8.851075
5.591765	5.851442	5.542585	5.508447	5.71852	5.571061	5.776745	5.897655	5.536492
7.019341	6.664379	6.825414	6.805993	7.074722	6.935466	7.022992	6.873911	7.011919
5.416968	5.35677	5.1895	5.504185	5.603343	5.28775	5.234006	5.322973	5.383905
10.7845	11.12558	11.08682	10.99911	10.66984	11.03154	10.74792	10.7845	10.99911
9.633596	9.490242	9.904655	9.655201	9.403644	9.323794	9.466184	9.3389	9.410272
5.788809	5.638551	5.564369	5.680268	5.644952	5.634937	5.465783	5.601494	5.527286
7.862607	7.350778	7.568214	7.142037	7.715544	7.229722	7.234225	7.807968	7.874759
8.654425	8.326538	8.324465	8.128844	8.322996	8.4668	8.415192	8.40938	7.672012
8.06723	7.986888	8.185298	7.611245	7.696437	8.079478	7.779597	7.730664	7.671494
5.716234	5.575031	5.437597	5.540856	5.599706	5.853322	5.633137	5.595998	5.711419
5.552493	5.691731	5.56938	5.941483	5.749714	5.85892	5.596822	5.661373	5.622479
5.812116	6.073284	6.24031	6.204148	6.076489	6.141454	6.137796	6.098744	6.169511
5.424731	5.351784	5.2487	5.356498	5.278769	5.267793	5.453572	5.475272	5.463744
6.542221	6.607356	6.657906	6.635826	6.602598	6.659378	6.645341	6.442731	6.685713
5.90205	5.849308	5.792754	5.978002	5.93786	5.811206	5.897655	5.877776	5.928039
6.990015	7.196183	6.909137	7.198851	7.026919	7.152645	7.133015	7.01518	7.023363
6.523149	6.658204	6.719246	6.583791	6.607036	6.780745	6.626039	6.682985	6.726472
8.213156	7.417032	7.712005	7.209524	7.669432	7.57566	6.953315	7.397644	7.570209

GSM1369[illegible]	GSM1369[illegible]	GSM1369[illegible]	GSM1369[illegible]	GSM1369[illegible]	GSM1369[illegible]	GSM1369[illegible]	GSM1369[illegible]	GSM1369[illegible]
7.240308	6.925896	7.092224	6.851303	6.97605	6.571101	6.153239	6.429112	6.96931
5.210888	5.277653	5.219097	5.246675	5.148839	5.201988	5.287477	5.259171	5.273033
9.266336	8.738524	8.813121	9.226729	7.922582	8.336191	7.958305	8.219681	8.854491
6.307607	6.231334	6.251029	6.329281	6.282715	6.2918	6.219105	6.285616	6.382241
7.268913	7.148911	7.221183	7.334343	7.379814	7.212365	7.049865	7.137728	7.223244
6.679502	6.329281	7.185092	6.695341	6.742568	6.570236	6.17082	6.182521	6.670528
5.22453	5.228395	5.253912	5.259709	5.170668	5.216937	5.386344	5.291777	5.394818
6.533197	5.845563	6.104532	6.181969	6.538331	6.089783	6.151661	6.163011	6.080184
5.914146	6.024901	5.879622	5.998686	5.970691	5.934571	5.98113	5.94488	5.951028
10.23848	10.60383	10.28346	10.51714	10.35389	10.55947	10.60669	10.4392	10.34354
5.621283	5.443341	5.618251	5.599425	5.578831	5.564917	5.525566	5.59282	5.550587
5.708962	5.65659	5.805014	5.676249	5.621283	5.674342	5.698937	5.74188	5.575031
11.74976	11.37274	12.06882	10.86586	11.62642	10.92398	10.6125	10.94077	11.19266
6.106211	6.347922	6.182521	6.24102	5.904047	6.262242	5.859682	6.250654	6.121075
5.367863	5.381617	5.384212	5.408945	5.119603	5.501836	5.74013	5.367236	5.537644
8.434187	8.02116	7.586678	7.969743	8.033876	7.726556	7.691453	7.840127	7.931779
6.757925	6.637935	6.682644	6.566639	6.632772	6.664129	6.790323	6.576192	6.564688
12.2125	12.39891	12.43078	12.43078	12.00752	12.41556	12.48865	12.28035	12.22437
6.565271	6.568765	6.526209	6.515746	6.567846	6.455263	6.503981	6.708785	6.611062
5.476295	5.500738	5.32929	5.406858	5.349619	5.487109	5.447353	5.462998	5.349066
7.619318	7.235392	7.848599	7.707397	7.714524	7.547698	7.267398	7.171625	7.470435
6.296239	5.763557	6.068396	6.293937	5.877022	5.657313	5.844122	5.891498	5.753214
7.527205	7.261338	7.300691	7.699778	7.338626	7.426478	7.263249	7.563035	7.428607
8.518731	8.90708	8.829743	8.718793	8.037364	8.793583	8.975705	8.801224	8.402778
7.87534	8.558284	8.523488	8.317559	8.415192	7.921559	8.055833	8.510293	7.996344
6.616619	6.255253	6.613532	6.41541	6.499488	6.339607	6.32241	6.295474	6.362935
9.273075	8.815754	8.927769	9.053994	9.046056	9.141065	8.839663	8.885597	9.125525
5.588955	5.652345	6.022753	5.768508	5.692495	5.833907	6.009866	5.749273	5.907357
6.63223	6.976243	7.102946	6.988884	6.893363	7.078071	6.977631	6.888128	6.866209
5.365717	5.270842	5.252209	5.300017	5.397099	5.243738	5.21726	5.193164	5.275188
10.57541	10.8497	10.20727	10.60669	11.03154	10.56612	10.91572	10.78819	10.65094
9.538848	9.408822	9.141065	9.467648	9.411684	9.329995	9.305659	9.503092	8.88749
5.696116	5.614974	5.576627	5.58946	5.582812	5.590742	5.674342	5.726989	5.763317
7.237693	7.654855	7.01865	8.104939	6.999223	7.239849	7.600845	7.124985	7.321451
7.583876	8.43567	8.481022	8.461831	8.227401	8.039119	8.580783	8.349365	8.341593
7.753363	7.92329	7.87363	7.618885	7.786265	7.839119	7.969203	7.899496	7.446295
5.847454	5.650067	6.075565	6.079602	5.75856	5.901083	5.594305	5.755737	6.029061
5.726748	5.740399	5.584143	5.614699	5.594875	5.524777	5.268629	5.497213	5.646744
6.305811	6.166114	6.038687	6.067182	6.034769	6.064255	6.236808	6.274647	6.096632
5.736589	5.497213	5.495879	5.478314	5.372253	5.40574	5.387698	5.315225	5.225291
6.685713	6.686023	6.468153	6.625491	6.545048	6.599712	6.516901	6.560368	6.592052
5.945858	5.807857	5.817451	5.881564	5.843918	5.910819	5.806154	5.721406	5.820063
7.148911	7.051939	6.700329	7.011919	6.826806	6.928991	6.80826	7.072144	6.916543
6.520596	6.573303	6.517673	6.43347	6.525912	6.502617	6.559821	6.482414	6.421599
7.377762	7.833983	7.771402	7.384773	7.221976	7.469185	7.745567	7.792327	7.874759
