# Supplementary material for: Identification of ferroptosis-associated biomarkers for the potential diagnosis and treatment of postmenopausal osteoporosis
Source: Front Endocrinol (Lausanne). 2022 Aug 29;13:986384. doi: 10.3389/fendo.2022.986384 (PMC9464919; doi:10.3389/fendo.2022.986384)
Supplement: Supplementary Table 3 — Identified 37 DEGs. [file DataSheet_3.pdf]

MGAT1
RALY
PLEC
STXBP2
LILRA6
CEBPA
GRINA
FXYD5
GPSM3
GNB2
CFP
FLNA
HGS
PLD3
JUNB
PTPN6
MAN2B1
RHOG
CTSD
H1FX
ZYX
ZFP36L2
TCIRG1
PKN1
EFHD2
CST3
FMNL1
STAB1
LIMD2
CSF3R
SPI1
NRGN
YWHAE
TLN1
LSP1
C3AR1
TMEM176A
