# Supplementary material for: Identification of ferroptosis-associated biomarkers for the potential diagnosis and treatment of postmenopausal osteoporosis
Source: Front Endocrinol (Lausanne). 2022 Aug 29;13:986384. doi: 10.3389/fendo.2022.986384 (PMC9464919; doi:10.3389/fendo.2022.986384)
Supplement: Supplementary file 1 [file boxplot.pdf › Original data an R codes 1/08.diff/boxplot.pdf]

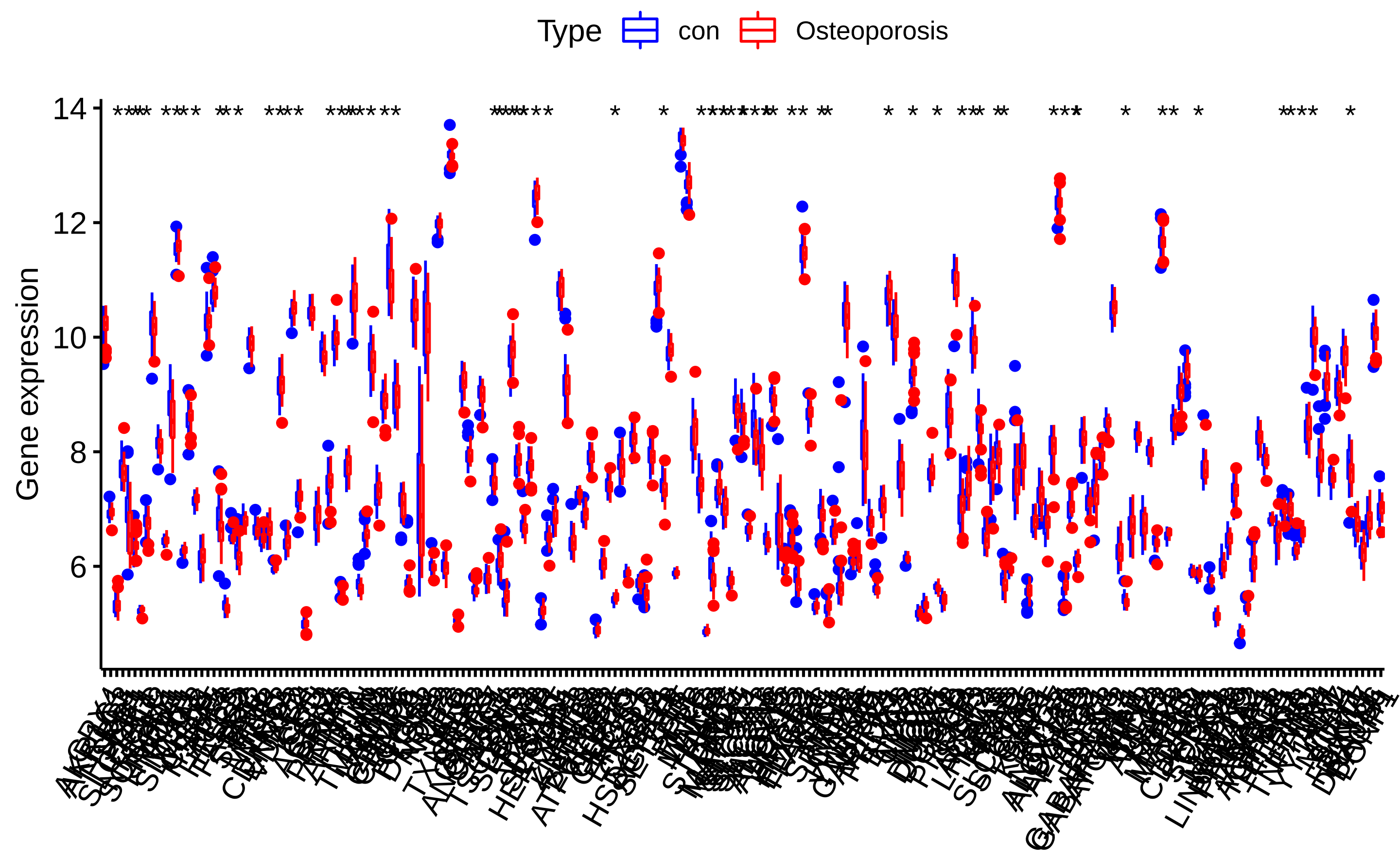
Type
con
Osteoporosis
Gene expression
14
12
10
8
6
